# Deepwater Chondrichthyan Bycatch of the Eastern King Prawn Fishery in the Southern Great Barrier Reef, Australia

**DOI:** 10.1371/journal.pone.0156036

**Published:** 2016-05-24

**Authors:** Cassandra L. Rigby, William T. White, Colin A. Simpfendorfer

**Affiliations:** 1 Centre for Sustainable Tropical Fisheries and Aquaculture & College of Marine and Environmental Sciences, James Cook University, Townsville, Queensland, 4811, Australia; 2 CSIRO Australian National Fish Collection, National Research Collections Australia, Hobart, Tasmania, 7001, Australia; Instituto Español de Oceanografía, SPAIN

## Abstract

The deepwater chondrichthyan fauna of the Great Barrier Reef is poorly known and life history information is required to enable their effective management as they are inherently vulnerable to exploitation. The chondrichthyan bycatch from the deepwater eastern king prawn fishery at the Swain Reefs in the southern Great Barrier Reef was examined to determine the species present and provide information on their life histories. In all, 1533 individuals were collected from 11 deepwater chondrichthyan species, with the Argus skate *Dipturus polyommata*, piked spurdog *Squalus megalops* and pale spotted catshark *Asymbolus pallidus* the most commonly caught. All but one species is endemic to Australia with five species restricted to waters offshore from Queensland. The extent of life history information available for each species varied but the life history traits across all species were characteristic of deep water chondrichthyans with relatively large length at maturity, small litters and low ovarian fecundity; all indicative of low biological productivity. However, variability among these traits and spatial and bathymetric distributions of the species suggests differing degrees of resilience to fishing pressure. To ensure the sustainability of these bycatch species, monitoring of their catches in the deepwater eastern king prawn fishery is recommended.

## Introduction

Deepwater chondrichthyans (i.e. shark, skate, ray and chimaera species whose distributions or majority of life cycle is predominantly below 200 m depth) are generally slower growing, later maturing and longer lived than chondrichthyans from shelf and pelagic habitats [1]. This reduces their biological productivity and capacity to recover from exploitation, rendering them typically more vulnerable to fishing pressure than chondrichthyans from other habitats [2, 3]. Information on the life history of many deepwater chondrichthyan species is lacking and is needed to enable assessment of their ability to sustain fishing pressure and for the development of effective conservation and management strategies [4].

The Great Barrier Reef Marine Park (GBRMP) has one third of its area in deepwater (>200 m depth) with these areas poorly known and rarely surveyed [5]. Most of the chondrichthyans reported from deep habitats within the GBRMP were collected in research trawl surveys by the FRV *Soela* in the mid 1980’s [6]. To date, 54 species of deepwater chondrichthyans are known to occur in the GBRMP [7] (S1 Table). Thirty-five of these are endemic to Australia with 19 of the endemics occurring only in waters offshore from Queensland, and a further eight species restricted to the east coast of Australia (S1 Table). This high level of endemism and geographically restricted distribution is common among deepwater chondrichthyans and potentially further reduces their resilience to fishing pressure [8].

There is a general lack of life history data on deepwater chondrichthyan species in the GBRMP with a number of the species listed as Threatened by the IUCN Red List of Threatened Species and almost half listed as Data Deficient (S1 Table). They are considered at risk in the GBRMP due to the lack of biological information, their intrinsic vulnerability and their capture as bycatch in commercial fisheries [9, 10]. A number of deepwater line fisheries and a trawl fishery operate within the GBRMP. The line fisheries are dispersed across the GBRMP with generally low and sporadic effort and deepwater chondrichthyans catches poorly reported in logbooks (partly exacerbated by problems with species identifications) and there are concerns about increasing effort and the effect on these deepwater species [11]. In contrast, the deepwater eastern king prawn (EKP) trawl fishery sector operates in a more limited area that includes the southern GBRMP around the Swain Reefs (~22°S) and waters further south outside the GBRMP to the New South Wales border (~28°S). Approximately half of the deepwater EKP fishery sector occurs within the GBRMP. This GBRMP area is exposed to high levels of trawling, with the seafloor trawled an average of 2.1 times in 2009 [10]. The Swain Reefs area is a poorly known shelf and upper continental slope habitat where any chondrichthyans present have been rated as being at high risk as a precaution due to the trawl effort and paucity of knowledge of their biology [10].

Around Swain Reefs, EKP fishers can trawl all year round in depths down to 250–300 m [12, 13], and tend to fish deeper in this area than further south. The EKP fishery is predominantly a night time fishery as the prawns are more active at night. It was difficult to define the number of boats and effort in the Swain Reefs area, as the EKP fishery also includes a shallow water sector (<90 m) and vessels within the EKP fishery, of which there were around 200 in 2014 [13], can fish in either sector. However, fewer boats tend to fish the deeper waters because it is further to travel and the gear is heavier with three wider nets deployed rather than two (for better stability), and more cable for the greater depths [12]. The annual effort in the northern part of the EKP fishery (from Fraser Island at ~26°S to Swain Reefs) was about 6,000 boat days in 2008–2011, with the majority of the deepwater EKP catch taken around Swain Reefs area [13]. The EKP trawl fishery deploys turtle excluder and bycatch reduction devices in the nets that generally exclude larger chondrichthyans (greater than about one metre in length) but have a minimal effect on reducing the catch of the smaller individuals and species which includes many of the deepwater chondrichthyans [12, 14, 15].

The chondrichthyan bycatch of the shallow water sector of the EKP fishery and the deepwater EKP sector further south around 27°S have been the focus of one previous study[12]. The species composition of the southern deepwater EKP sector was based on one research trawl trip that was for a duration of ten nights and that fished at depths of 110–165 m [12]. The only information on chondrichthyan bycatch of the deepwater EKP sector around Swain Reefs is from one trip by the Department of Agriculture and Fisheries Observer Program that was for a duration of nine nights [10]. Information on the bycatch of deepwater chondrichthyans and their life histories in this little known Swain Reefs area of the deepwater EKP sector is required to ensure their sustainability.

Given the need for better data on deepwater chondrichthyans to help improve management, the present study aimed to provide information on the species composition and biology of chondrichthyans captured in the poorly known northern part of the deepwater EKP fishery. This will improve knowledge of the species encountered in the deeper waters of the GBRMP and advance information on the life history of these species. It will also facilitate management to more confidently assess the risks of the deepwater trawl fishery to the southern GBRMP.

## Materials and Methods

### Ethics statement

Sampling was conducted under Queensland Department of Agriculture and General Fisheries Permits (No. 55105 & 147714) and Great Barrier Reef Marine Park Authority Permit (No. G10/33603.1). All procedures were approved by James Cook University’s Animal Ethics Committee (no. A1566, 1933).

### Sampling

The chondrichthyan bycatch of the deepwater EKP fishery around the Swain Reefs was observed on two commercial prawn trawlers during their normal trawling activities. On each vessel a five-week trip was undertaken: 1 June–6 July 2011 and 14 March–18 April 2012. Demersal trawl fishing gear comprised three otter trawl nets of 15 fathoms each (i.e. head rope length of 27 m) with cod end meshes of 44.5 mm and Turtle Excluder and Bycatch Reduction Devices. Trawling was from dusk till dawn with each trawl shot in one direction. The date, time, depth (m) and latitude and longitude (WGS 84) of the start and end of each shot were recorded. The start and end of each shot was taken from when the trawl nets reached and left the seafloor, respectively.

All deepwater chondrichthyans captured during the two, five week trips were deceased upon landing on the vessel and were identified, sexed and labelled. They were snap frozen whole, retained and upon completion of each trip were transported frozen to the laboratory where they were stored frozen until processed. Any shelf sharks and rays landed were recorded, photographed and returned to the sea, with the majority alive when returned (S2 Table). Fisheries Queensland, Department of Agriculture and Fisheries donated some deepwater chondrichthyan specimens retained from their commercial deepwater EKP fishery observer surveys at Swain Reefs.

### Specimen identification

All chondrichthyans were identified by taxonomic features at sea using the keys in Last and Stevens [16]. A tissue sample (fin clip) was collected from a subsample of specimens of each deepwater chondrichthyans species for molecular species identification. The samples were analysed as part of an ongoing National Science Foundation project: Chondrichthyan Tree of Life, led by the College of Charleston in the United States. The methodology for these molecular analyses sequenced for the mitochondrial NADH2 gene is described in [17]. Representative specimens of each deepwater species were lodged as voucher specimens at the Australian National Fish Collection (CSIRO, Hobart).

### Deepwater chondrichthyan processing

All specimens were thawed, sexed, weighed (*M*_T_) (± 0.1 g) and measured (± 1 mm) following Francis [18]: stretched total length (*L*_ST_) and fork length (*L*_F_) for sharks; total length (*L*_T_) for skates and stingarees; disc width (*W*_D_) for stingrays; and body length (*L*_C_—snout to posterior end of supracaudal fin), precaudal length (*L*_PC_—snout to anterior edge of supracaudal fin) and snout to vent length (*L*_SV_) for chimaeras. Differences in the sex ratio were tested by Chi-square test with Yates’ correction. Where samples sizes were sufficient, the relationships between *L*_ST_ and *L*_F_ and *L*_ST_ and *W*_D_ were examined using linear regression.

Specimens were dissected to remove ageing structures (dorsal fin spines and vertebrae) and to investigate their reproductive biology. The ageing structures were cleaned and sectioned and a number of stains trialled to enhance vertebral growth band visibility, that is, Alizarin red S [19], crystal violet and silver nitrate [20], cobalt nitrate [21, 22], graphite powder [23], ninhydrin [24], nitric acid [25], and Mayer’s haematoxylin (modified technique of Bubley *et al*. [26]. More detailed descriptions of the ageing methodology are presented in [27, 28]. Reproductive staging of all species was adapted from Ebert [29] and Walker [30]. Males were classed immature (claspers pliable and shorter than pelvic fins), adolescent (claspers extended past the pelvic fins but still pliable), and mature (claspers extended past the pelvic fins and were rigid and fully calcified, testes were developed and epididymides were coiled). The presence of sperm in the epididymides was noted. Females were classed as immature (undifferentiated ovaries, undeveloped oviducal glands and thin uteri), adolescent (developing ovaries with white follicles, developing oviducal glands, slightly expanded uteri), and mature (yolked follicles, fully developed oviducal gland and uteri). For those species with sufficient sample sizes, estimates of population length at 50% maturity (*L*_ST50_) with 95% confidence intervals were determined for males and females separately using a generalised linear model with a binomial error structure and logit-link function within the statistical package ‘R’ [31]. For other species the range of length at maturity was reported. The *L*_ST50_ or mid-point of the range of length at maturity were used to determine the life history invariant ratio of relative length at maturity (*L*_ST50_/ *L*_ST_) [32]. For the invariant ratios, where the range of length of maturity was large, the length of maturity from the literature was used and the maximum length of the males and females from the literature was used when it was greater than that sampled in this study.

Reproductive systems were removed and left and right testes and ovaries (including epigonal organs) weighed separately (*M*_G_) (± 0.1 g). The number of yolked follicles in each ovary and the maximum follicle diameter (*D*_Fmax_) (± 1 mm) were recorded. The number of yolked follicles can be used as a measure of ovarian fecundity; a proxy for fecundity as egg laying rates of oviparous species are difficult to define [8]. Where there were sufficient data, relationships between the total length and total ovary weight (*M*_G_), *D*_Fmax_ and number of yolked follicles were examined by least squares linear regression. When present, the number of embryos and the sex (if able to be determined by visual inspection of presence/absence of claspers), presence of internal or external yolk, uterus (left or right), total length (± 0.1 mm) and mass (± 0.1 g) of each embryo were noted. When present, the number of egg cases and uterus (left or right) and mass (± 0.1 g) were noted and egg case length (*L*_EC_) (± 0.1 mm) taken following Ebert and Davis [33]. All egg cases were labelled, frozen and retained. Any recently born elasmobranchs were noted and could be mostly distinguished as neonates by the presence of an umbilical/yolk sac scar on their ventral body surface in the region between the pectoral fins.

## Results

### Sampling

**Fig 1:**
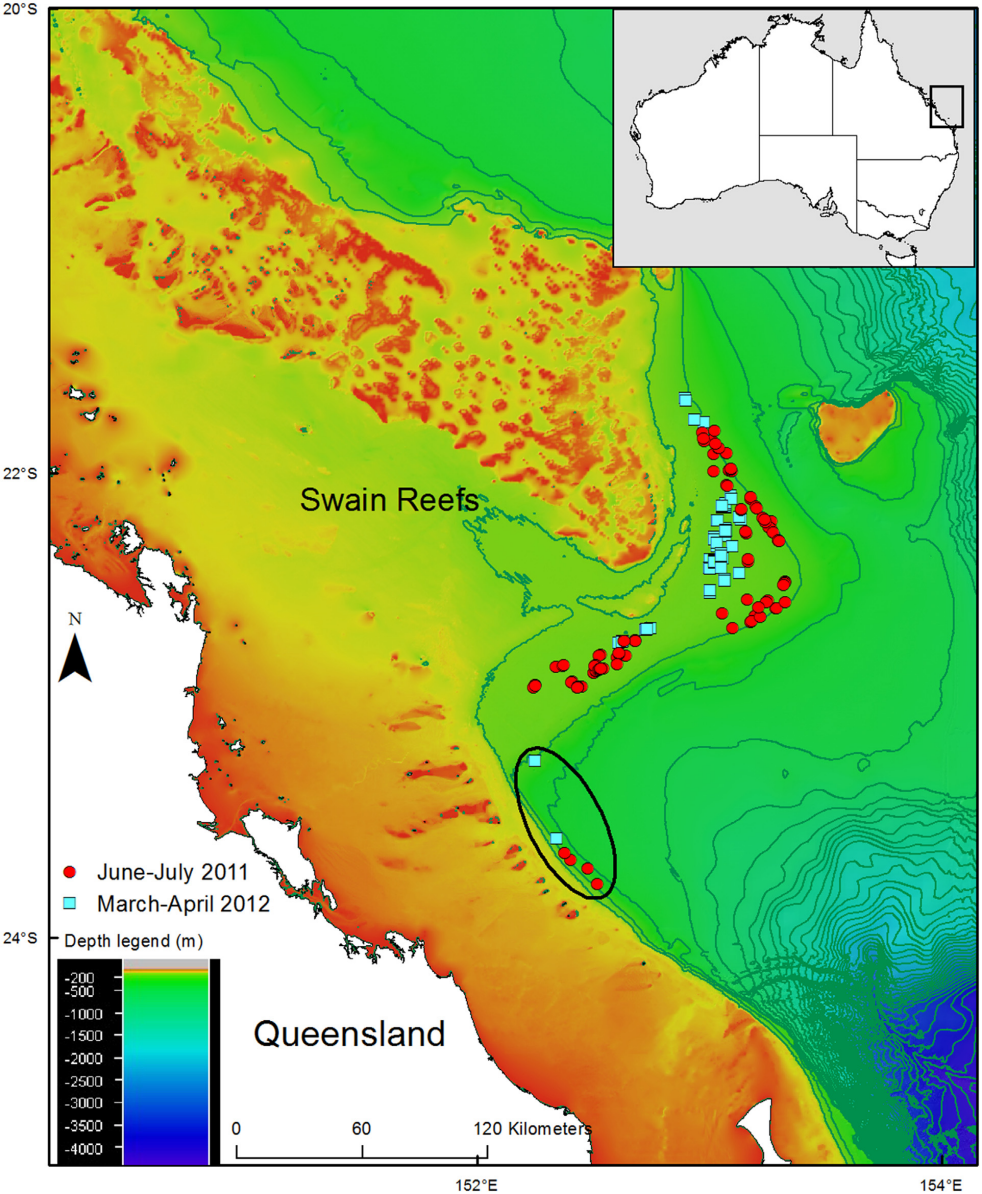
Sample locations for the chondrichthyan bycatch in the deepwater eastern king prawn fishery around Swain Reefs. The depth contours are at 100 metre intervals. The six shots south of main trawl ground are circled.

A total of 211 trawl shots were observed across a depth range of 117–280 m (Fig 1). The majority of the trawls were between 150–200 m on the shelf in the main deepwater EKP fishery trawl grounds around Swain Reefs with six shots in deeper waters (203–280 m) to the south of the main trawl grounds (Fig 1). Deepwater chondrichthyans were observed in 72% of the trawl shots. There was an average of four shots per night, though in rough weather trawling ceased and over the two, five weeks trips 105 shots were observed on Trip 1 and 106 shots on Trip 2. The trawl shots had an average duration of 2.5 hours and speed of 5.4 kmh^-1^.

### Species composition

A total of 1533 individuals were observed from eleven species of deepwater chondrichthyans (Table 1). Some shelf species of sharks and rays were also observed, but were far less abundant with a total of 142 individuals from thirteen species recorded (S2 Table). Argus skate *Dipturus polyommata* dominated the bycatch of deepwater species by number (50.1%; Table 1). Along with the next two most dominant bycatch species, piked spurdog *Squalus megalops* and pale spotted catshark *Asymbolus pallidus*, these three species accounted for 92.3% by number of the deepwater bycatch (Table 1).

**Table 1:** Observed deepwater chondrichthyan species by sex, abundance and length. The animals were collected from 211 trawl shots during June–July 2011 and March–April 2012.

Scientific name	Common name	Male (n)	Female (n)	Total (n)	Depth range (m)	Length range (mm)	% deep-water bycatch
*Dipturus polyommata*	Argus skate	366	402	768	135–280	95–371	50.1
*Squalus megalops*	Piked spurdog	117	305	422	187–280	253–505	27.5
*Asymbolus pallidus*	Pale spotted catshark	110	115	225	174–280	141–436	14.7
*Mustelus walkeri*	Eastern spotted gummy shark	14	34	48	124–242	410–1050	3.1
*Urolophus piperatus*	Coral sea stingaree	10	8	18	123–216	158–367	1.2
*Hydrolagus lemures*	Blackfin ghostshark	8	9	17	203–242	465–820 *L*_C_	1.1
*Urolophus bucculentus*	Sandyback stingaree	4	10	14	159–242	175–690	0.9
*Squatina albipunctata*	Eastern angelshark	4	7	11	132–242	510–1160	0.7
*Dipturus apricus*	Pale tropical skate	5	3	8	237–280	177–279	0.5
*Cephaloscyllium variegatum*	Saddled swellshark	0	1	1	215	675	0.1
*Pristiophorus delicatus*	Tropical sawshark	0	1	1	176	949	0.1
Total abundance deepwater species				1533			

All lengths are for *L*_ST_ or *L*_T_ unless otherwise specified as chimaera length (*L*_C_). The percent of deepwater bycatch is by abundance.

**Fig 2:**
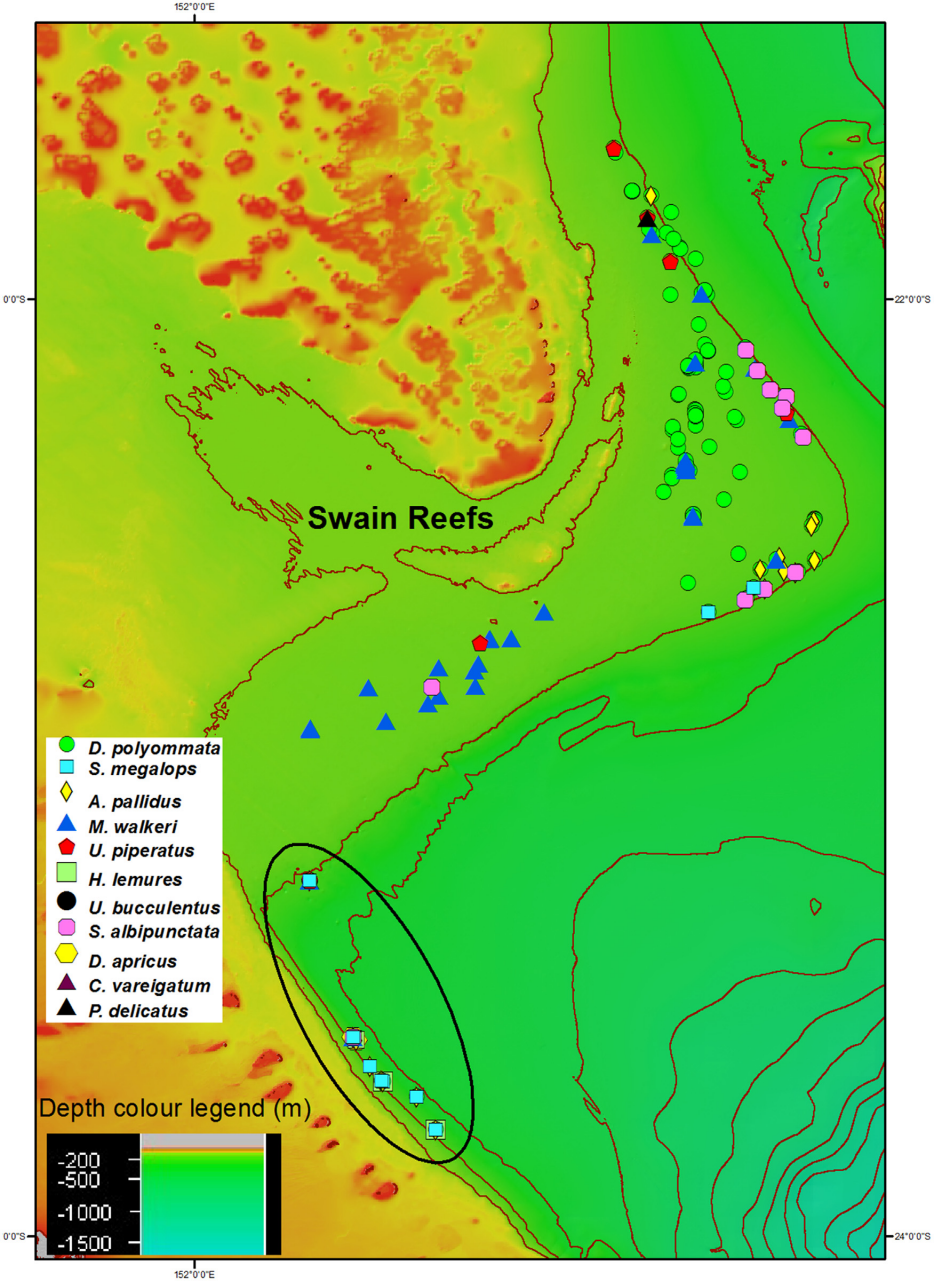
Distribution of deepwater chondrichthyans in the eastern king prawn deepwater fishery around Swain Reefs. The six shots south of main trawl ground are circled and all deepwater species sampled occur in these six shots, except *Pristiophorus delicatus*.

The results from this study added four new records of deepwater species to the known chondrichthyan fauna of the deepwater EKP fishery: *S*. *megalops*, coral sea stingaree *Urolophus piperatus*, eastern angelshark *Squatina albipunctata* and tropical sawshark *Pristiophorus delicatus*. This study also added *Squalus megalops* to the known deepwater species in the GBRMP (S1 Table). In addition, this study found it is likely that all previously recorded patchwork stingaree *Urolophus flavomosaicus* (morphological identification) were actually the sandyback stingaree *Urolophus bucculentus* (molecular identification; see Specimen Identification section and S1 and S3 Tables). This likely revision of *U*. *flavomosaicus* to *U*. *bucculentus* extended the northern distribution of *U*. *bucculentus* from Stradbroke Island, Queensland (27°35’) to the Swain Reefs (21°41’ S) [16]. The single *P*. *delicatus* collected was outside the current recorded distribution for the species, extending the known northern limit slightly, from off Rockhampton (22°10 S’) to the Swain Reefs (21°49’ S). This individual was caught in 207 m (in the north of the trawl grounds, Fig 2), 38 m shallower than the previously recorded upper depth limit of 245 m [16, 34]. The individual was 960 mm TL, 120 mm longer than any specimen of *P*. *delicatus* previously recorded (840 mm) [16]. Two other species had their upper depth limit extended by this study: *A*. *pallidus* from 225 m to 174 m and *U*. *piperatus* from 171 m to 123 m [35, 36].

### Specimen identification

The molecular analyses confirmed the morphological identifications of *S*. *megalops*, *A*. *pallidus*, *U*. *piperatus*, *S*. *albipunctata*, pale tropical skate *Dipturus apricus* and saddled swellshark *Cephaloscyllium variegatum* (S3 Table). The NADH2 sample results will be available on the Chondrichthyan Tree of Life project website by mid-2016 [37]. The NADH2 sequences for *D*. *polyommata* (S3 Table) were almost identical with those for its sister species Endeavour skate *Dipturus endeavouri* from further south in Queensland [37]. The *Mustelus walkeri* sequences (S3 Table) were almost identical with those of gummy shark *Mustelus antarcticus* in southern Australia [37]. The sequences of blackfin ghostshark *Hydrolagus lemures* (S3 Table) were identical to samples from Western Australia, Tasmania, the Tasman Sea and New South Wales which have been identified as both *H*. *lemures* and Ogilby’s ghostshark *Hydrolagus ogilbyi* [37]. The identifications of these three species, *D*. *polyommata*, *M*. *walkeri* and *H*. *lemures*, were retained based on the explanations provided in the Identification section of the Discussion.

The specimens of a reticulated *Urolophus* were identified in the field as patchwork stingaree *Urolophus flavomosaicus* based on their colour pattern, which is the only distinguishing taxonomic feature between *U*. *flavosmosaicus* and *U*. *bucculentus* [16]. The sequences from specimens of this reticulated *Urolophus* (S3 Table) grouped close to but distinct from *U*. *flavomosaicus* from Western Australia. These sequences were identical to a sequence from a *U*. *bucculentus* specimen collected off New South Wales, and thus the specimens were identified as *U*. *bucculentus* with further comments on this made in the Discussion [37]. The single specimen of *Pristiophorus delicatus* was morphologically identified with a high degree of confidence by a trained identifier and was registered in the Australian National Fish Collection.

### Distribution

The majority of deepwater chondrichthyans were taken in the six trawl shots to the south of the main trawl grounds that were all in waters > 200 m deep (Fig 2). The only species caught regularly throughout the main trawl grounds was *D*. *polyommata*. Other species also present in the main trawl grounds but caught infrequently were *M*. *walkeri*, *S*. *albipunctata*, *U*. *piperatus*, and *P*. *delicatus*. Most of the *S*. *megalops* and *A*. *pallidus* were taken south of the main trawl grounds. Those individuals that were present in the trawl grounds (7% of the total number of *S*. *megalops* and 12% of total number of *A*. *pallidus*) all occurred in the same area. This area was at the edge of the shelf on the eastern extremity of the deepwater EKP fishery area sampled (Fig 2). All the deepwater chondrichthyans observed as bycatch are endemic to Australia, except *S*. *megalops*, with five of the ten endemics only occurring in waters offshore from Queensland (S4 Table). All the Queensland endemics have a wider distribution than the Swain Reefs deepwater EKP fishery area (S4 Table).

### Biological aspects

The extent of biological data varied greatly between the deepwater species collected. Three species had adequate sample sizes and reliable age structures that enabled age, growth and reproductive studies: *D*. *polyommata*, *S*. *megalops* and *M*. *walkeri*. The studies for these three species were detailed and are published in [27, 28], with the data summarised in Table 2 and S5 Table. Preliminary biological data were available for *A*. *pallidus*, *U*. *piperatus*, *U*. *bucculentus*, *H*. *lemures* and *S*. *albipunctata* and are described below and summarised in Table 2. Of these five species, only *A*. *pallidus* had sufficient numbers of mature animals to enable estimates of population length at 50% maturity (*L*_ST50_). No biological data was available for *D*. *apricus*, *C*. *variegatum* or *P*. *delicatus* due to the small number of individuals collected from each of these three species (Table 1).

**Table 2:** Biological data for deepwater chondrichthyans from the eastern king prawn deepwater fishery around Swain Reefs. The animals were collected from 211 trawl shots during June–July 2011 and March–April 2012.

	Max. length (mm)		Length at maturity (mm)		Relative length at maturity		Length at birth (mm)	Litter size	Ovarian fecundity
	Male	Female	Male	Female	Male	Female			
*D*. *polyommata*^a^	369	371	278 *L*_T50_	303 *L*_T50_	0.73	0.80	89–111	NA	7.6 (mean)
*S*. *megalops*^b^	407	505	352 *L*_ST50_	422 *L*_ST50_	0.81	0.84	157–158	2.5 (mean)	
*M*. *walkeri*^a^	805	1050	670–805	833–1012	0.92	0.83	273–295	5–7	
*A*. *pallidus*	428	436	330 *L*_ST50_	352 *L*_ST50_	0.75	0.75	~140^c^	NA	8.5 (mean)
*U*. *piperatus*	289	367	205–273	233–366	0.48	0.56	NA	3^d^	
*U*. *bucculentus*	507	690	<447	300–466	0.62	0.53	N	2–4	
*H*. *lemures*	620 *L*_C_	820 *L*_C_	500–533 *L*_C_	625–718 *L*_C_	0.83	0.82	NA	NA	3–11
*S*. *albipunctata*	706	1160	NA	720–1160	NA	0.82	NA	4^e^	
*D*. *apricus*	279	232	NA	NA	NA	NA	NA	NA	
*C*. *variegatum*	NA	675	NA	NA	NA	NA	NA	NA	
*P*. *delicatus*	NA	949	NA	NA	NA	NA	NA	NA	

All lengths are for *L*_ST_ or *L*_T_ or unless otherwise specified as chimaera length (*L*_C_). Length at maturity are range estimates unless stated as *L*_ST50_ = estimates of population length at 50% maturity. Relative length at maturity is (*L*_ST50_/ *L*_ST_). Length at birth are range estimates, unless noted otherwise. Litter size and ovarian fecundity are range estimates, unless otherwise noted or stated as mean. *L*_C_ = chimaera length. NA = no information available from the individuals collected in this study.

Source:

^a,b^ [28];

note:

^c^estimated from one neonate collected,

^d^only two pregnant individuals collected, both with 3 embryos,

^e^only one pregnant individual collected.

### Asymbolus pallidus

**Fig 3:**
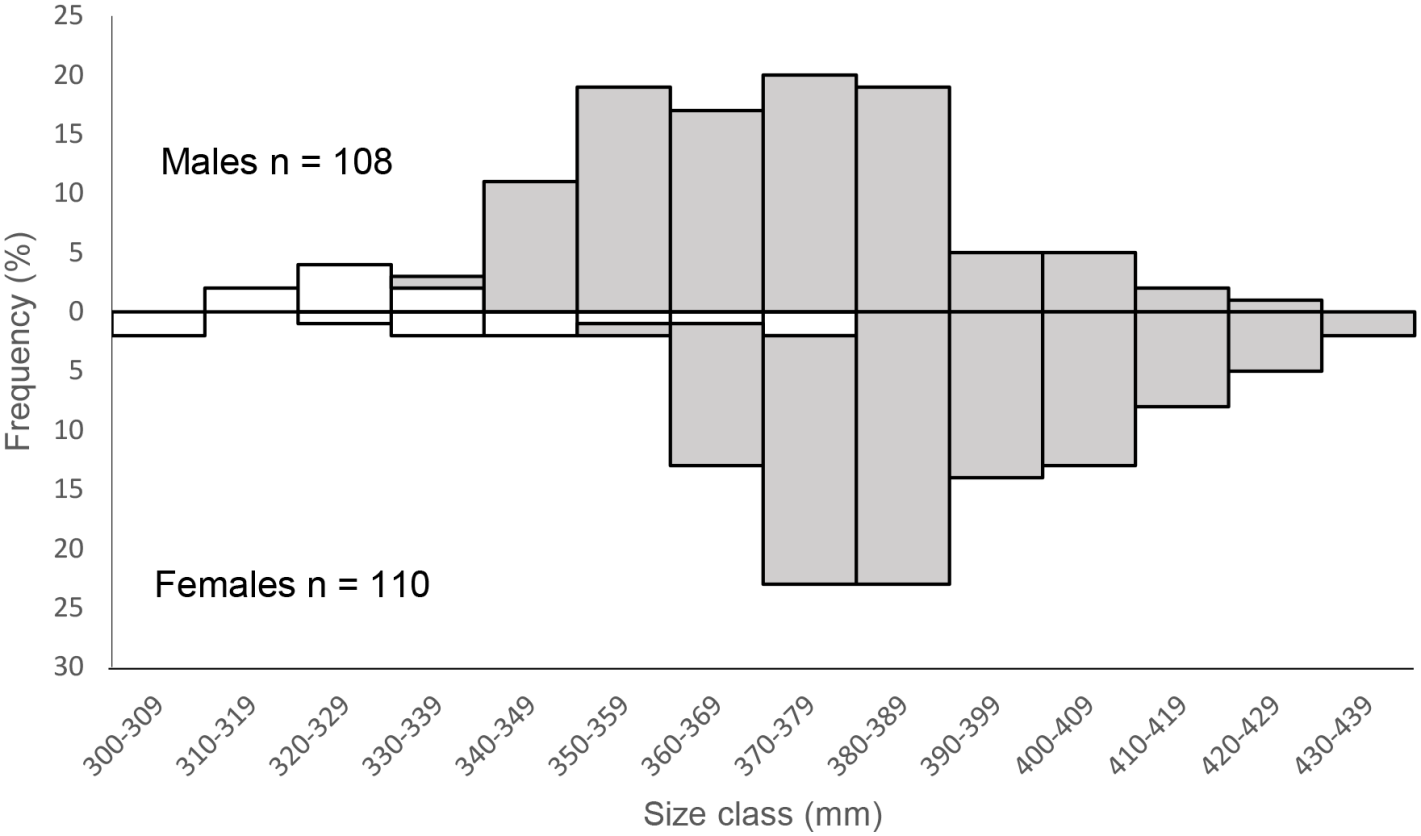
Length-frequency of immature (white) and mature (grey) *Asymbolus pallidus* individuals.

The size range of male *A*. *pallidus* collected was 314–428 mm *L*_ST_ and for females was 141–436 mm *L*_ST_) (Tables 1 and 2). The sex ratio across all trawls was not significantly different from parity (χ^2^ = 2.76, d.f = 1, p = 0.10). Fork length was not measured on this species due to the elongated shape of the caudal fin. Two males and three females were lodged as voucher specimens at the Australian National Fish Collection (CSIRO) with the remainder dissected. The specimens were dominated by mature males and females (Fig 3).

**Fig 4:**
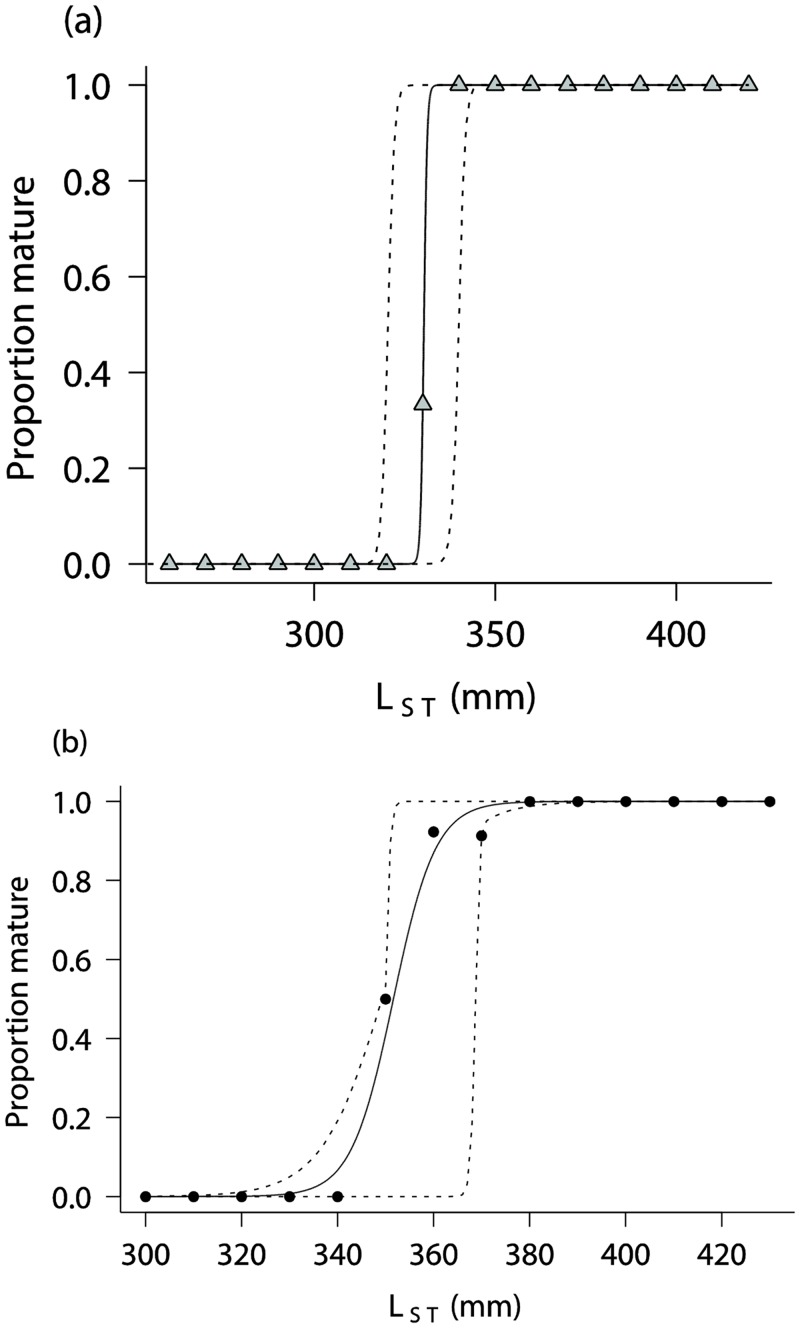
Length at maturity ogives for *Asymbolus pallidus*. (a) males and (b) females. Dashed lines are 95% confidence intervals.

Although there were sufficient numbers of *A*. *pallidus* for an age study, the species had no reliable ageing structure as growth bands were not discernible on either whole or sectioned vertebrae and it had no other hard parts for ageing. Trials with the different stains failed to enhance vertebral growth band visibility. Length at maturity data was available for 108 males with all males mature at ≥ 339 mm. The estimate of *L*_ST50_ (Table 2, Fig 4) was very similar to the reported value of 320 mm [16]. The length at maturity data was available for 110 females and indicated that the smallest mature female was 351 mm and the largest immature female 371 m. Combined with the estimate of *L*_ST50_ (Table 2, Fig 4), this is the first length at maturity data available for females of this species. The invariants (Table 2) were calculated with the reported maximum length of 439 mm and 467 for males and females, respectively [36].

**Fig 5:**
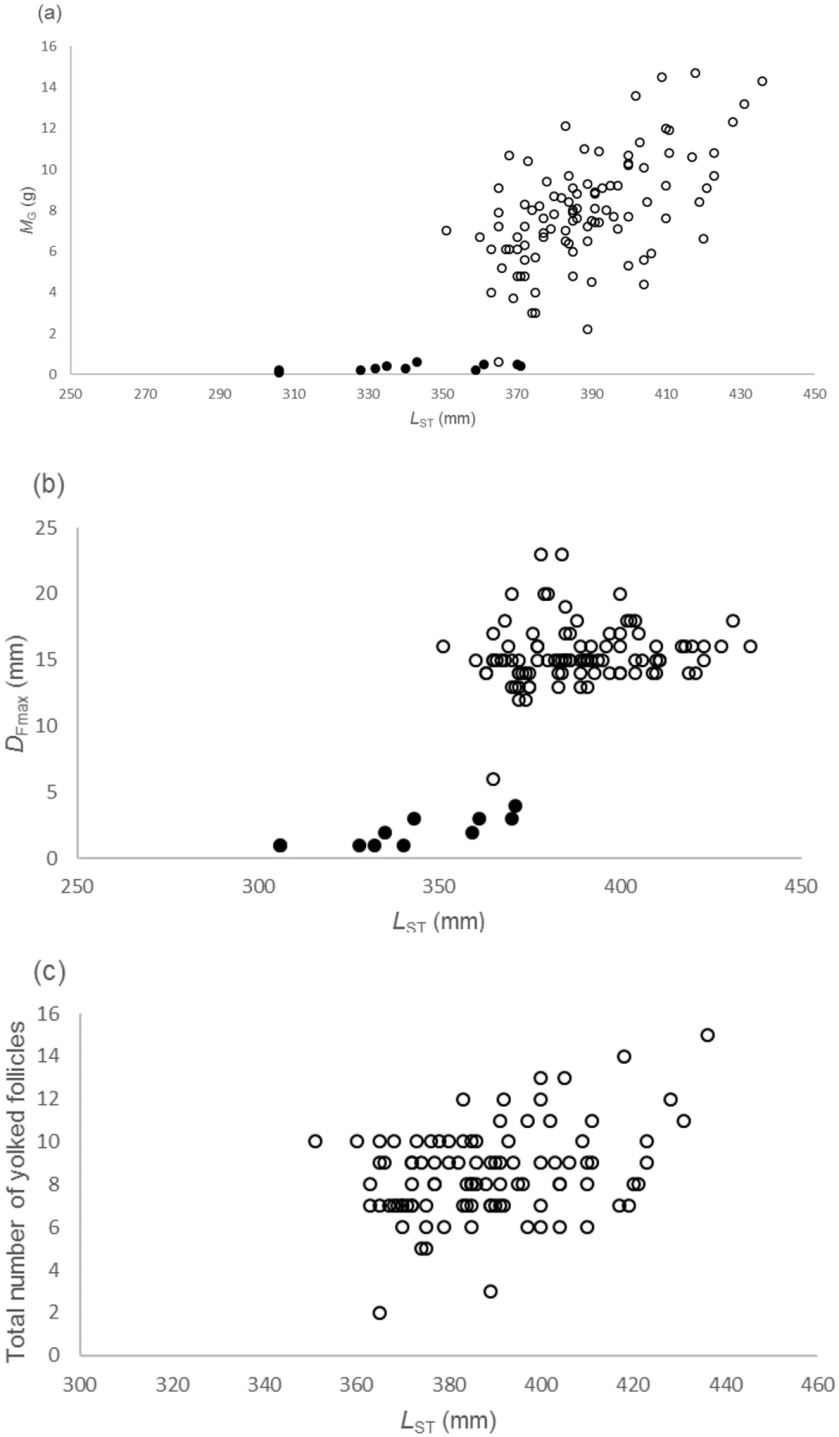
The relationship between stretched total length (*L*_ST_) and reproductive indices of *Asymbolus pallidus* females. (a) *L*_ST_ and total ovary weight (*M*_G_), (b) *L*_ST_ and maximum follicle diameter (*D*_Fmax_) and (c) *L*_ST_ and number of yolked follicles. Immature (closed circle) and mature (open circle).

Only the right ovary was functional as reported for other catsharks [38, 39]. In mature females the mean ± se total number of yolked follicles in the right ovary was 8.5 ± 0.2 (range 2–15). The yolked follicles ranged in diameter from 4–23 mm. The ovary weight (*M*_G_) and maximum follicle diameter *D*_Fmax_ increased rapidly after maturity (Fig 5). In mature females there were significant relationships between *M*_G_ and *L*_ST_: *M*_G_ = 0.09 *L*_ST_− 25.42 (*R*^2^ = 0.33, D.f. = 98, p < 0.001) but the relationship was not significant for *D*_Fmax_ and *L*_ST_: *D*_Fmax_ = 0.017 *L*_ST_ + 8.55 (*R*^2^ = 0.02, D.f. = 98, p = 0.16). The number of yolked follicles increased significantly with length in mature females: number of follicles = 0.04 *L*_ST_− 7.06 (*R*^2^ = 0.12, d.f. = 98, p < 0.001) (Fig 5).

**Fig 6:**
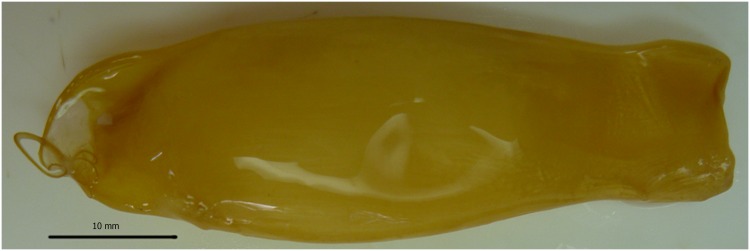
Egg case from a 423 mm stretched total length *Asymbolus pallidus*. Anterior end at left of image (49.3 mm egg case length).

Females with egg cases *in utero* were collected on both sampling trips with a total of 22 collected; the smallest gravid female was 366 mm *L*_ST_. The proportion of mature females that were gravid was similar on both trips: 22% and 25% on trips 1 and 2, respectively. Gravid females were not segregated as they were collected in the same trawl shots as immature and mature non-gravid females and immature and mature males. The majority of the 22 gravid females were in depths >200 m (203–280 m) south of the main trawl grounds with only 4 gravid females collected in the trawl grounds at depths < 200 m, i.e. at 174–196 m. All 22 gravid females had 6–10 yolked follicles in the right ovary with a diameter of 5–23 mm, and an egg case in each uterus. The egg cases were golden (Fig 6) and ranged in length from 44.6–51.5 mm *L*_EC_ and 2.5–4.0 g; there were no visible embryos within any of the egg cases. A free swimming female neonate of 141 mm *L*_ST_ was collected from 215 m depth south of the main trawl ground. The neonate had been feeding, evidenced by fish scales and a vertebra in the stomach. This extends the length of hatching from the previously reported 190 mm [16] to 140–190 mm *L*_ST_ (Table 2).

### Urolophus piperatus

A total of 18 *U*. *piperatus* was collected with similar numbers of males and females: 10 males (205–289 mm *L*_T_) and 8 females (158–367 mm *L*_T_) (Table 2). The relationship between *L*_ST_ and *W*_D_ for sexes combined was: *L*_T_ = 1.52 *W*_D_− 15.19 (*R*^2^ = 0.98, d.f. = 17, p <0.001). The smallest male was immature and captured in the main trawl grounds at 192 m. The remainder of males (273–289 mm *L*_T_) were mature with sperm present in the epididymides and were all captured in one trawl shot to the south of the main fishing grounds in 215 m. Hence, males mature between 205–273 mm *L*_T_ which encompassed the reported length at maturity of 230 mm [16].

Five of the females were immature (158–233 mm *L*_T_) and three mature, two of which were pregnant and one *post partum* (366–367 mm *L*_T_). Hence, females mature between 233–366 mm *L*_T_ (Table 2) which includes the known length of maturity of 270 mm based on one female [16, 35]. The life history invariants of relative length at maturity were 0.48 and 0.56 for males and females, respectively (Table 2). The maximum length (484 mm *L*_T_) and the length at maturity were used from the literature [16, 35].

The two pregnant females were caught in two consecutive trawl shots in the trawl grounds in 177 m. The *post partum* female was also caught in the trawl grounds at 129 m. Only the left ovary and uterus were functional, which is typical for urolophids [40]. All three females had yolked follicles in the left ovary (n = 5, 6, 10) with a diameter of 5–15 mm and an expanded left uterus thickened with villi. One female aborted two embryos upon capture and retained one embryo (1 male, 2 female) and upon capture the other female aborted all three embryos (2 male, 1 female) (Table 2). All embryos had external yolk sacs attached and were 50.0–72.9 mm *L*_T_ and 1.2–3.3 g.

### Urolophus bucculentus

A total of 14 *U*. *bucculentus* was collected with more females than males: 4 males (447–507 mm *L*_T_) and 10 females (175–690 mm *L*_T_) (Table 2). The relationship between *L*_T_ and *W*_D_ for sexes combined was: *L*_T_ = 1.20 *W*_D_ + 21.51 (*R*^2^ = 0.99, d.f. = 12, p <0.001). All males were mature and captured in one trawl shot to the south of the main fishing grounds in 215 m, where they occurred together with females of all life cycle stages. All mature males had sperm present in the epididymides. The life history invariant of relative length at maturity was 0.62 (Table 2) and was calculated using the length at maturity (414 mm) and maximum length of males (672 mm) from the literature [40]

Six of the females were immature (175–300 mm *L*_T_) and four mature, two of which were pregnant and two *post partum* (466–690 mm *L*_T_). Females mature between 300–466 mm *L*_T_ (Table 2). The females in this study mature at a smaller length than the previously reported 478–522 mm (*L*_T50_ = 502 mm) [40]. The life history invariant of relative length at maturity was 0.53 (Table 2) and was calculated using the minimum sampled length at maturity from this study (466 mm) and maximum length of females (885 mm) from the literature [40].

The pregnant and *post partum* females were caught in depths above and below 200 m, in the main trawl grounds and in the area to the south of these grounds, respectively. None of the pregnant or *post partum* females had yolked follicles in the left ovary and all had an expanded left uterus thickened with villi. Both females aborted upon capture, one aborted two embryos (1 male, 1 female) and the other four embryos (2 male, 2 female) (Table 2). All embryos had external yolk sacs attached and were 63.0–98.0 mm *L*_ST_ and 2.4–9.1 g.

### Hydrolagus lemures

A total of 17 *H*. *lemures* was collected with similar numbers of males and females: 8 males (465–620 *L*_C_) and 9 females (480–820 *L*_C_) (Table 2). The combined sexes relationship for: *L*_C_ and *L*_PC_ was: *L*_C_ = 1.05 *L*_PC_ +35.47 (*R*^2^ = 0.99, d.f. = 13, p <0.001); and for *L*_C_ and *L*_SV_ was: *L*_C_ = 1.55 *L*_SV_ + 173.03 (*R*^2^ = 0.98, d.f. = 13, p <0.001). This study extended the maximum recorded length from 530 mm to 820 mm *L*_C_ [16]. Two males were immature at 465–500 mm *L*_C_ and the rest mature (all with sperm present) (533–620 mm *L*_C_), hence males mature between 500–533 mm *L*_C_ which concurs with the reported 500 mm [16]. Six females were immature (480–540 mm *L*_C_), one adolescent (625 mm *L*_C_) and two mature (718–820 mm *L*_C_), hence females mature at 625–718 mm *L*_C_ (Table 2). This is the first length at maturity data for females of this species. The mature females had yolked follicles in the right and left ovaries (n = 3–11) with a diameter of 10–36 mm.

The invariant relative lengths at maturity were similar for males and females (0.83 and 0.82 respectively, Table 2) and were calculated with the midpoint of the lengths of maturity and maximum lengths of the males and females from this study. All 17 individual *H*. *lemures* were collected in the area south of the main trawl grounds. Within this southern area, the immature females were caught in the same location as the males at 203–280 m. The adolescent and mature females were separate from the males in a different location, and were all caught together in the one trawl shot at 242 m.

### Squatina albipunctata

A total of 11 *S*. *albipunctata* was collected with more females than males: 4 males (592–706 mm *L*_ST_) and 7 females (510–1160 mm *L*_ST_) (Table 2). The relationship between *L*_ST_ and *L*_F_ for sexes combined was: *L*_ST_ = 1.03 *L*_F_ + 16.12 (*R*^2^ = 0.98, d.f. = 9, p <0.001). All males were immature and taken in the trawl grounds on the shelf edge at 190–196 m. Six of the females were immature (510–720 mm *L*_ST_) and caught in the trawl grounds with the immature males at depths of 190–196 m, except the smallest female that was caught south of the main trawl grounds in 238 m. The one mature female (1160 mm *L*_ST_) was pregnant and was caught on its own, in the trawl grounds at a shallower depth (133 m) than all other *S*. *albipunctata* encountered. Hence the female length at maturity is 720–1160 which includes the known female length at maturity of 1070 mm [16]. The relative length at maturity was 0.82 (Table 2) calculated from the literature maximum length of 1300 mm and known length of maturity [16]. Only the left ovary appeared functional which is the case in some, but not all species of *Squatina* [41]. The ovary had numerous small follicles, but none were yolked. There were four embryos (Table 2), two in the left uterus and two in the right uterus. They all had external yolk sacs and were too small to sex at 49.5–54.7 mm *L*_ST_ and 0.8–1.7 g.

## Discussion

This study increased the known number of deepwater chondrichthyan species recorded from the deepwater EKP fishery around the Swain Reefs. The bycatch community of sympatric deepwater chondrichthyans in this area of the fishery displayed life history traits typical of deepwater chondrichthyans [1, 2]. However, there was some variability evident in these life history traits among the EKP deepwater chondrichthyans and in their patterns of depth and spatial distribution. Three species of chondrichthyans had sufficient sample sizes and reliable ageing structures for age and growth studies: two shark species (*S*. *megalops* and *M*. *walkeri*) and a skate (*D*. *polyommata)* [27, 28]. The *S*. *megalops* traits were typical of deepwater dogfish as it grew slowly, was long lived and had small litters [27]. The *M*. *walkeri* had a faster growth rate than the *S*. *megalops*, but in comparison to other *Mustelus* species it grew more slowly and matured later, which was probably a reflection of the greater depth of occurrence than most *Mustelus* [28]. The *D*. *polyommata* traits were mostly typical of small-medium bodies skates as it had a moderately fast growth rate, which was faster than that of the two shark species, and it also had a shorter life span and younger age at maturity than the two shark species [27, 28]. It did, however have a low ovarian fecundity relative to that of other *Dipturus* species [28] The preliminary biological data from the other species indicated they had relatively large lengths at maturity, small litters, low ovarian fecundity and in some cases non-continuous reproductive cycles which are a combination of traits suggestive of low productivity [42].

### Identification

The molecular analyses of tissues from specimens of four deepwater chondrichthyan species (*D*. *polyommata*, *M*. *walkeri*, *H*. *lemures* and *U*. *bucculentus*) raised some taxonomic questions which were addressed and resolved in different ways for each of the species. The *D*. *polyommata* sequence data suggested they are very close to *D*. *endeavouri*. The two species, however differ in colour pattern and subtle morphology [43], and are possibly only relatively recently separated from one another. The *M*. *walkeri* molecular data did not distinguish them from *M*. *antarcticus* using the NADH2 gene, however these two species differ in some morphological features [44] and life history traits [28]. One of the morphological features that separates these two species, the extent of the buccopharyngeal denticles on the roof and floor of the mouth [44], was examined in all *M*. *walkeri* specimens collected in this study. In all examined specimens, the buccopharyngeal denticles covered the entire floor and palate of the mouth, which is distinctive of *M*. *walkeri*. *Mustelus antarcticus* only has these denticles on the anterior third to half of the floor and palate [44]. Furthermore, extensive tagging work indicated that *M*. *antarcticus* does not extend into Queensland waters [30]. The key morphological characteristics of the *H*. *lemures* specimens recorded in this study aligned well with those provided by Last and Stevens [16] for *H*. *lemures* rather than *H*. *ogilbyi*. This species belongs to a poorly defined *Hydrolagus lemures-ogilbyi* species complex which needs a detailed taxonomic investigation to resolve. The *Urolophus* specimens obtained in this study possessed the complex pattern of reticulations and large white spots typical of *U*. *flavomosaicus*, but not over the central disc region. The central disc area of these specimens more closely resembled that of typical *U*. *bucculentus* specimens, i.e. small white spots and fine reticulations [16] and the sequence data was identical to *U*. *bucculentus* [37]. Thus, we consider these specimens to be conspecific with *U*. *bucculentus* and not *U*. *flavomosaicus*. Further taxonomic investigation is required to elucidate whether *U*. *flavomosaicus* records from eastern Australia are actually just a northern colour variant of *U*. *bucculentus*, with these being sister species.

### Sexual dimorphism and life history invariants

Sexual dimorphism, where the females were larger and had a length at maturity larger than the males, was apparent in all of the deepwater chondrichthyans collected in this study and is typical of many chondrichthyans [4, 45]. Dimorphism is known among urolophids [40, 46, 47], chimaerids [48, 49] and squatinids [41, 50, 51]. Dimorphism has been attributed to the need for females to partition more time and energy into growth before reproductive age is reached to be of sufficient length to support the production of relatively large young [52, 53]. This dimorphism has been reported in other small-medium bodied skates closely related to *D*. *polyommata*, (such as whitespotted skate *Dipturus cerva* and long-nose skate *Dipturus confusus* [54]), in populations of *S*. *megalops* that occur in other parts of Australia and elsewhere [55], and in other *Mustelus* species [28, 30, 56].

Among the catsharks, length at maturity is often similar for both sexes [38], though the dimorphism of *A*. *pallidus* has also been noted for the closely related orange spotted catshark *Asymbolus rubiginosus* that occurs further south in Queensland, and in some other catshark species [57–60]. The length at maturity of *A*. *pallidus* males and females was similar to that of another small sized catshark New Zealand catshark *Bythaelurus dawsoni* [59].

The relatively large invariant length at maturity was evident for nearly all the deepwater species sampled. It reflects a relatively large length for onset of breeding and is typical of deepwater chondrichthyans for which the mean invariant length at maturity across all deepwater taxa has been reported as 0.76 and ranged to 0.90 [1, 38, 61, 62]. The exceptions were the two urolophids, *U*. *piperatus* and *U*. *bucculentus*. The only other urolophid present in deepwater, the wide stingaree *Urolophus expansus*, has a typical deepwater length at maturity invariant of 0.74 [63], hence the relatively smaller length at maturity for the female *U*. *bucculentus* may be a reflection of the smaller length at maturity reported for this northern population compared to that from southern Australian waters [40]. The smaller invariant of *U*. *piperatus* may be attributed to the small sample size used from the literature to assess maturity and more samples may be required [35].

### Reproduction

All the deepwater chondrichthyans sampled that were able to provide biological data had low biological productivity characteristic of chondrichthyans from deep habitats [2, 63]. They had a combination of reproductive traits that varied from long cycles and small litters to shorter continuous reproductive cycles (where yolked follicles are present in pregnant females) with low fecundity. The viviparous species all had small litters and included both continuous and non-continuous cycles. The *S*. *megalops* litter sizes and continuous reproduction was similar to that previously reported for this species that is known to have a long biennial cycle [55]. The only other species for which the reproductive cycle was formerly known, *U*. *bucculentus*, also has a long biennial cycle, with the non-continuous reproduction and litter sizes observed in this study concurred with that previously described [40]. *Squatina albipunctata* appeared to have a non-continuous reproductive cycle with litter sizes similar to those cited before, and also likely has a longer biennial cycle, as this has been reported for the three other deepwater *Squatina* species [41, 51, 64]. *Mustelus walkeri* and *U*. *piperatus* both appear to have a continuous cycle that may be annual as the cycles were annual in other species of their genera [30, 40]. The litters of *M*. *walkeri* were smaller than the closely related *M*. *antarcticus* and other aplacental *Mustelus* species, although were only based on two pregnant females so should be interpreted with caution [28, 30]. The smaller litter size may be attributed to the likely deeper depths inhabited by *M*. *walkeri* compared to other *Mustelus* species, with greater depths reported to be associated with smaller litter sizes as potentially less energy is available in deeper waters to invest in reproduction and growth [1]. The *U*. *piperatus* litter size was typical of many deepwater and small sized *Urolophus* species, which mostly have small litters of only 2 to 5 [16, 40, 47].

The two gravid oviparous species, *D*. *polyommata* and *A*. *pallidus* both appeared to have a continuous reproductive cycle. Year round oviposition is common among skates and has previously been reported for other *Dipturus* species [54, 65, 66]. *Dipturus polyommata* was the least fecund of the *Dipturus* species for which ovarian fecundity data was available; *D*. *endeavouri*, *D*. *cerva* and *D*. *confusus* [54, 65]. *Dipturus polyommata* was smaller than the two latter southern Australian species, which could account for the lower fecundity, but was of similar size to *D*. *endeavouri*. The smallest yolked follicle in *D*. *polyommata* was 5 mm in diameter [28], whereas it was 3 mm in diameter in *D*. *endeavouri* [65]; the larger sized follicles may reduce the total number of mature follicles that can occur in similar sized body. However, the reason for different mature yolk sizes in these recently taxonomically separated sister species is not known and cannot be postulated without definitive data on their depth and geographic ranges.

This study confirmed *A*. *pallidus* as single oviparous (one egg case per uterus), similar to its close relatives which also show year round oviposition, though the proportion of females carrying egg cases was lower than reported in other species of catsharks [57, 59, 67]. The *A*. *pallidus* ovarian fecundity was also lower than that of two close relatives further south in Queensland [57]. However, these relatives were both larger bodied and the *A*. *pallidus* fecundity was similar to another small sized deepwater catshark, *B*. *dawsoni* [59]. The other oviparous species *H*. *lemures* was not gravid and although the ovarian fecundity range was only from two females, it was similar to that of other deepwater chimaerids of similar size for which ovarian fecundity has been reported; spotted ratfish *Hydrolagus colliei* and rabbitfish *Chimaera monstrosa* [48, 49].

Although all these deepwater bycatch species generally are likely to have low productivity, they present a variety of reproductive types and lengths of cycles that likely lead to varying degrees of resilience. This was apparent even within a genus, for e.g. both urolophids had low fecundity but *U*. *piperatus* likely has an annual cycle whereas *U*. *bucculentus* is biennial and so is probably less resilient to fishing pressure as a biennial cycle lowers productivity [55]. This highlights the need for species to be individually assessed to accurately determine their productivity and susceptibility to commercial fishing.

### Fishery effects

The majority of deepwater chondrichthyans taken as bycatch in this study were caught outside the main shelf trawl grounds. While the Swain Reefs EKP fishery is generally on the shelf, there is some fishing in the deeper waters down to 250–300 m depth on the upper slope habitat, particularly when prawn abundances are lower on the shelf and fishers move deeper searching for prawns. On the two trips observed for this study, the prawns were in abundance on the shelf area and hence that is where the vessels mainly fished. Consequently, the deepwater chondrichthyan bycatch recorded in this study may not be fully representative of all species that occur across the entire area of the deepwater EKP fishery that can be fished. Although the sampling was from two trips in different months and years, there was unlikely any direct seasonal effect on the deepwater chondrichthyan species composition as water temperature and salinity at depths of 100–2000 m fluctuate little within and between years in the vicinity of Swain Reefs [68]. However, there may have been an indirect effect due to a large mesoscale eddy, the ‘Capricorn Eddy’ which forms just south of Swain Reefs, predominantly in September–November but also in June-August, that increases the availability of nutrients and food [69, 70]

The recorded species may also have been influenced by trawl gear length selective sampling bias. This could have affected both the lengths of species collected and the species composition as some larger deepwater chondrichthyans may have been excluded by the Turtle Excluder and Bycatch Reduction Devices. These generally allow animals over 1000 mm in length to escape the net [14, 15]. Trawling at night may also have influenced the bycatch species composition; many of the deepwater chondrichthyans occur to much greater depths than those trawled but were present in the shallower shelf waters. It is possible that some of the species may make diel vertical migrations, moving along the seafloor into shallower waters at night as a feeding strategy; a migration reported for the deepwater southern dogfish *Centrophorus zeehani* [71] and some other deepwater squaloid sharks [8]. Finally, it is also possible that the deepwater bycatch species composition in the area fished has been impacted by the fishery over time. The deepwater EKP fishery in the Swain Reefs area has operated since around 1997 and less resilient species are likely to have been more greatly affected than those species with higher biological productivity.

*Dipturus polyommata* dominated the bycatch on the shelf area. Skates are susceptible to capture by trawl gear [72] and a closely related skate, *D*. *endeavouri* was also the most dominant species in the bycatch of the deepwater EKP fishery further south in Queensland [12]. The fishery around Swain Reefs interacted with all life stages of *D*. *polyommata*, from newly hatched juveniles to mature and gravid adults with no sexual, maturity or length segregation evident. This lack of spatial segregation has been observed in temperate Australian *Dipturus* species and is common among other skates [29, 54, 73]. Although this increases the potential impact of the fishery on *D*. *polyommata*, of all the deepwater chondrichthyans encountered, *D*. *polyommata* may be more resilient to fishing pressure as it grows moderately fast, has moderate longevity and a continuous reproductive cycle [28]. This combination of traits in other small skates has been linked to increased resilience to fishing pressure [74]. *Dipturus polyommata* also has refuge in deeper waters and to the north of the Swain Reefs, as the fishery is at the southern edge of the species’ range.

There was also a lack of segregation apparent for *S*. *megalops*, the other dominant bycatch species. Strong sexual segregation has been reported for temperate populations of *S*. *megalops* [75] but in this study catches were dominated by females of all stages of sexual maturity, and these females occurred together with immature and mature males, mostly south of the main trawl grounds [27]. *Squalus megalops* is an opportunistic predator [76] and the large mixed schools may have been due to the presence of the ‘Capricorn Eddy’ and its associated increased food availability. We hypothesis that an abundance of food and the small size of adult *S*. *megalops* may reduce the predatory threat to juveniles of their own species and allow for mixed schools [77, 78]. Among the other species sampled, nearly the full size range of most species was collected across the two sampling times, suggesting no bathymetric segregation by size or sex. This may be due to possible diel vertical migrations and the limited number of sampling trips because bathymetric segregation by size and sex has been reported as often occurring in deepwater sharks and chimaeras [8].

With respect to segregation, *Asymbolus pallidus* was an exception as the bycatch consisted mostly of adults with limited numbers of immature specimens captured, and other than the neonate, no animals <314 mm *L*_ST_ taken. Two other species of catshark, including *A*. *rubiginosus*, were dominant in the bycatch of the deepwater EKP fishery further south where there was also a lack of immature specimens [12, 57]. Gear selectivity was discounted as the cause of the lack of small individuals in the southern study, as the small trawl mesh should retain small sharks [57]. This suggests that immature animals of the species of catsharks taken across the deepwater EKP fishery may occupy deeper waters than adults [57]. The absence of young catsharks in trawls has also been attributed to their migration up into the water column after birth until they are larger, when they then return to the demersal habitat [38]. Despite mostly adult *A*. *pallidus* being captured in the EKP fishery; this species also has a refuge in deeper waters. Although it has a restricted distribution in offshore waters of Queensland, it also has refuge to the north of Swain Reefs because, similar to *D*. *polyommata*, the fishery is at the southern edge of the species range.

The other three species with distributions restricted to waters offshore of Queensland, *P*. *delicatus*, *M*. *walkeri* and *U*. *piperatus* all have refuge outside the fishery both spatially and at depth and were also all infrequently caught. However, the *M*. *walkeri* life history traits of slow growth, late maturity, high longevity and small litters are associated with a reduced capacity to recover from exploitation [2, 3, 28]. The *U*. *piperatus* also likely has low productivity because despite the continuous and possibly annual reproductive cycle, litters were small and high abortion rates on capture were evident. Abortion upon capture is typical of urolophids and can lead to reduced population viability [40, 46]. The likely low productivity of these deepwater chondrichthyans highlights the need to monitor catches of these species within the trawl fishery.

Two of the other deepwater chondrichthyans encountered that have broader Australian distributions, *S*. *albipunctata* and *U*. *bucculentus* are listed as Vulnerable on the IUCN Red List of Threatened species due to heavy fishing pressure in the southeast Australia and documented declines in abundance [79–81]. *Squatina albipunctata* has depth refuge from the deepwater EKP fishery and was caught infrequently however, this study has shown they have small litters and likely a non-continuous breeding cycle which indicates low biological productivity. The depth distribution of *U*. *bucculentus* (65–265 m) does not provide refuge from the Swain Reefs deepwater EKP trawl fishery. This study extended the known distribution of *U*. *bucculentus* from southern Queensland further north to Swain Reefs and while it has not been previously reported from the deepwater EKP fishery (either at the Swain Reefs or further south), *U*. *flavomosaicus* has been recorded, which was the field identification of *U*. *bucculentus* in this study. This study provides some evidence that *U*. *flavomosaicus* may not occur in eastern Australia, with the patterned form present in Queensland probably a colour variant of *U*. *bucculentus*. As *U*. *bucculentus* also occurs on the shelf in shallower depths it may be exposed to fishing pressure from other sectors of the Queensland East Coast Otter Trawl Fishery. However, it has not yet been recorded as a bycatch of the fishery, nor has *U*. *flavomosaicus* [10, 12].

The EKP fishery (shallow and deep) has been the focus of a recent biological and economic management strategy evaluation study [13]. The study was on the biology of the eastern king prawn and economics of the fishery with little discussion on the fishery bycatch. However, options for spatial and temporal closures and caps on total fishing effort were explored that could have ramifications for the deepwater chondrichthyan bycatch, that is, potentially providing a benefit through management of fishing pressure. A closure in part of the deepwater northern EKP area was considered but implementation not recommended as analyses indicated no benefit to yield or catch value [13]. The study proposed continuation of an EKP fishery steering committee to enable more effective assessment and implementation of management measures in the fishery. This could provide a forum for consideration of measures to ensure the sustainability of the deepwater chondrichthyan bycatch. Any proposed research and monitoring component of the EKP fishery management could include prioritisation of the deepwater chondrichthyans with respect to those species likely to be most vulnerable to fishing pressure, and promote support for better recording of such species through logbooks, onboard electronic monitoring techniques and observers. There is an implicit need for species-specific life history studies of some of these potentially more vulnerable species. Support for research of new methods that may reduce the catch of deepwater chondrichthyans would also be of benefit. For example, a recent study in a North Atlantic demersal finfish trawl fishery at depths of 120–170 m reported decreased catch rates of squaloid sharks, skates and rays by removing the tickler chain deployed on the footrope of the trawl gear [82].

This study has increased the knowledge of the life history of deepwater chondrichthyans taken as bycatch of the Swain Reefs deepwater EKP fishery and present in the southern GBRMP. The species all have life history traits typical of deep habitats and indicative of low biological productivity, though variability in their life history and distributions result in varying degrees of resilience to fishing pressure. This highlights the need for each species to be individually assessed for risk from the fishery. More information is required on the catches of some species to ensure their sustainability. As such, ongoing monitoring of the deepwater chondrichthyans species taken as bycatch within the deepwater EKP fishery is recommended.

## Supporting Information

S1 Table Deepwater chondrichthyans known to occur in the Great Barrier Reef Marine Park. Source: A. Great Barrier Reef Marine Park (Chin et al. 2010); B. Eastern King Prawn fishery in the Great Barrier Reef, Department of Agriculture and Fishery Observer Program 2005–2010(Pears et al. 2012); C. Eastern King Prawn fishery in the Swain Reefs area of the Great Barrier Reef (the present study). Australian endemic: if endemic it is stated if restricted to waters offshore from Queensland-New South Wales (QLD-NSW) or Queensland (QLD). IUCN Status from the IUCN Red List of Threatened Species http://www.iucnredlist.org.
(DOCX)

S2 Table Observed shelf shark and ray species by sex, abundance and length. All lengths are for *L*_ST_ unless otherwise specified as disc width (*W*_D_). The percent of shelf bycatch is by abundance.
(DOCX)

S3 Table Genetic samples for deepwater chondrichthyan specimens collected from Swain Reefs Eastern King Prawn Fishery. The GN numbers refer to samples with NADH2 sequences available as part of the Chondrichthyan Tree of Life project (http://sharksrays.org/).
(DOCX)

S4 Table Depth and distribution range of deepwater chondrichthyans observed from Swain Reefs Eastern King Prawn Fishery. Australian endemic: if endemic it is stated if restricted to waters offshore from Queensland-New South Wales (QLD-NSW) or Queensland (QLD). Source: Last and Stevens, 2009.
(DOCX)

S5 Table Age and growth data for *Dipturus polyommata*, *Squalus megalops* and *Mustelus walkeri*. The growth completion rate *k* is the von Bertalanffy growth function. Note: *M*. *walkeri* male age at maturity was based on the largest adolescent male and the one mature male collected. Source: Rigby *et al*. 2105, Rigby *et al*. 2016.
(DOCX)

## References

[1] Rigby C, Simpfendorfer CA. Patterns in life history traits of deep-water chondrichthyans. Deep Sea Research Part II: Topical Studies in Oceanography. 2015;115:30–40. 10.1016/j.dsr2.2013.09.004

[2] García VB, Lucifora LO, Myers RA. The importance of habitat and life history to extinction risk in sharks, skates, rays and chimaeras. Proc R Soc B. 2008;275(1630):83–9. 10.1098/rspb.2007.1295 17956843 PMC2562409

[3] Simpfendorfer C, Kyne PM. Limited potential to recover from overfishing raises concerns for deep-sea sharks, rays and chimaeras. Environ Conserv. 2009;36(02):97–103. 10.1017/S0376892909990191

[4] Cailliet GM, Goldman KJ. Age determination and validation in chondrichthyan fishes In: Carrier JC, Musick JA, Heithaus MR, editors. Biology of sharks and their relatives. Boca Raton, FL: CRC Press; 2004 p. 399–447.

[5] GBRMPA. Great Barrier Reef Outlook Report 2009. GBRMPA Townsville. 2009 [updated 8 September 20121 June 2015]. Available from: http://www.gbrmpa.gov.au/outlook-for-the-reef/great-barrier-reef-outlook-report.

[6] Last PR, Pogonoski JJ, Gledhill DC, White WT, Walker CJ. The deepwater demersal ichthyofauna of the western Coral Sea. Zootaxa. 2014;3887(2):191–224. 10.11646/zootaxa.3887.2.4 25543931

[7] Chin A, Kyne PM, Walker TI, McAuley RB. An integrated risk assessment for climate change: analysing the vulnerability of sharks and rays on Australia's Great Barrier Reef. Glob Change Biol. 2010;16(7):1936–53. 10.1111/j.1365-2486.2009.02128.x

[8] Kyne PM, Simpfendorfer CA. Deepwater chondrichthyans In: Carrier JC, Musick JA, Heithaus MR, editors. Sharks and their relatives II Biodiversity, adaptive physiology, and conservation. Boca Raton, Florida: CRC Press; 2010 p. 37–113.

[9] GBRMPA. Great Barrier Reef Outlook Report 2014. GBRMPA Townsville 2014 [1 June 2015]. Available from: http://www.gbrmpa.gov.au/outlook-for-the-reef/great-barrier-reef-outlook-report.

[10] Pears RJ, Morison AK, Jebreen EJ, Dunning MC, Pitcher CR, Courtney AJ, et al. Ecological risk assessment of the East Coast Otter Trawl Fishery in the Great Barrier Reef Marine Park: Summary report. Townsville: 2012.

[11] Sumpton W, McLennan M, Campbell M, Kerrigan B. Assessing technology changes and risks to the sustainable management of deepwater line fisheries in southern Queensland. Department of Agriculture, Fisheries and Forestry. FRDC Project 2010/053., 2013.

[12] Courtney AJ, Haddy JA, Campbell DP, Roy DP, Tonks ML, Gaddes SW, et al. Bycatch weight, composition and preliminary estimates of the impact of bycatch reduction devices in Queensland's trawl fishery. Report to the Fisheries Research Development Corporation No. 2000/170, 2007.

[13] Courtney AJ, O'Neill MF, Braccini JM, Leigh GM, Kienzle M, Pascoe A, et al. Biological and economic management strategy evaluations of the eastern king prawn fishery. FRDC Final Report April 2014. Report to the Fisheries Research Development Corporation No. 2008/019, 2014.

[14] Brewer D, Heales D, Milton D, Dell Q, Fry G, Venables B, et al The impact of turtle excluder devices and bycatch reduction devices on diverse tropical marine communities in Australia's northern prawn trawl fishery. Fish Res. 2006;81(2–3):176–88. 10.1016/j.fishres.2006.07.009

[15] Courtney AJ, Campbell MJ, Tonks ML, Roy DP, Gaddes SW, Haddy JA, et al Effects of bycatch reduction devices in Queensland's (Australia) deepwater eastern king prawn (*Melicertus plebejus*) trawl fishery. Fish Res. 2014;157(0):113–23. 10.1016/j.fishres.2014.03.021

[16] Last PR, Stevens JD. Sharks and rays of Australia. 2nd ed Melbourne: CSIRO Publishing; 2009 644 p.

[17] Naylor GJP, Caira JN, Jensen K, Rosana KAM, White WT, Last PR. A DNA sequence–based approach to the identification of shark and ray species and its implications for global elasmobranch diversity and parasitology. Bull Am Mus Nat Hist. 2012:1–262. 10.1206/754.1

[18] Francis M. Morphometric minefields—towards a measurement standard for chondrichthyan fishes. Environ Biol Fishes. 2006;77(3):407–21. 10.1007/s10641-006-9109-1

[19] Officer RA, Gason AS, Walker TI, Clement JG. Sources of variation in counts of growth increments in vertebrae from gummy shark, (*Mustelus antarcticus*, and school shark, *Galeorhinus galeus*): implications for age determination. Can J Fish Aquat Sci. 1996;53(8):1765–77. 10.1139/f96-103

[20] Schwartz FJ, editor Shark ageing methods and age estimation of scalloped hammerhead, *Sphyrna lewini*, and dusky, *Carcharinus obscurus*, sharks based on vertebral ring counts. Proceedings of the international workshop on age determination of oceanic pelagic fishes: tunas, billfishes, and sharks; 1983.

[21] Hoenig JM, Brown CA. A simple technique for staining growth bands in elasmobranch vertebrae. Bull Mar Sci. 1988;42:334–7.

[22] Gennari E, Scacco U. First age and growth estimates in the deep water shark, *Etmopterus spinax* (Linnaeus, 1758), by deep coned vertebral analysis. Mar Biol. 2007;152(5):1207–14. 10.1007/s00227-007-0769-y

[23] Ferreira BP, Vooren CM. Age, growth, and structure of vertebra in the school shark *Galeorhinus galeus* (Linnaeus, 1758) from Southern Brazil. Fish Bull. 1991;89(1):19–31. ISI:A1991FE84000003.

[24] Davenport S, Stevens J. Age and growth of two commercially important sharks (*Carcharhinus tilstoni* and *C*. *sorrah*) from northern Australia. Mar Freshw Res. 1988;39(4):417–33. 10.1071/MF9880417

[25] Correia JP, Figueiredo IM. A modified decalcification technique for enhancing growth bands in deep-coned vertebrae of elasmobranchs. Environ Biol Fishes. 1997;50(2):225–30. 10.1023/a:1007368025164

[26] Bubley WJ, Kneebone J, Sulikowski JA, Tsang PCW. Reassessment of spiny dogfish *Squalus acanthias* age and growth using vertebrae and dorsal-fin spines. J Fish Biol. 2012;80(5):1300–19. 10.1111/j.1095-8649.2011.03171.x 22497385

[27] Rigby CL, Daley RK, Simpfendorfer CA. Comparison of life histories of two deepwater sharks from eastern Australia: the piked spurdog and the Philippine spurdog. Marine and Freshwater Research. 2015.

[28] Rigby CL, White WT, Smart JJ, Simpfendorfer CA. Life histories of two deep-water Australian endemic elasmobranchs: Argus skate *Dipturus polyommata* and eastern spotted gummy shark *Mustelus walkeri*. J Fish Biol. 2016;88:1149–74. 10.1111/jfb.12891 26806022

[29] Ebert DA. Reproductive biology of skates, *Bathyraja* (Ishiyama), along the eastern Bering Sea continental slope. J Fish Biol. 2005;66(3):618–49. 10.1111/j.0022-1112.2005.00628.x

[30] Walker TI. Spatial and temporal variation in the reproductive biology of gummy shark *Mustelus antarcticus* (Chondrichthyes: Triakidae) harvested off southern Australia. Mar Freshw Res. 2007;58(1):67–97. 10.1071/MF06074

[31] R Development Core Team. R: A language and environment for statistical computing. R Foundation for statistical computing Vienna, Austria: 2014.

[32] Dulvy NK, Forrest RE. Life histories, population dynamics and extinction risks in chondrichthyans In: Carrier JC, Musick JA, Heithaus MR, editors. Sharks and their relatives II Biodiversity, adaptive physiology, and conservation. Boca Raton, Florida: CRC Press; 2010 p. 639–79.

[33] Ebert DA, Davis CD. Descriptions of skate egg cases (Chondrichthyes: Rajiformes: Rajoidei) from the eastern North Pacific. Zootaxa. 2007;1393:1–18.

[34] Yearsley GKL, P.R, White WT. A new species of sawshark, *Pristiophorus delicatus* sp. no. (Pristiophoriformes: Pristiophoridae), from northeastern Australia In: Last PR, White WT, Pogonoski JJ, editors. Descriptions of new Australian Chondrichthyans. No. 022. Hobart: CSIRO Marine and Atmospheric Research; 2008 p. 23–33.

[35] Seret B, Last P. Description of four new stingarees of the genus *Urolophus* (Batoidea: Urolophidae) from the Coral Sea, South-West Pacific. Cybium. 2003;27(4):307–20.

[36] Last PR, Gomon MF, Geldhill DC. Australian spotted catsharks of the genus *Asymbolus* (Carcharhiniformes: Scyliorhinidae). Part 2: Descriptions of three new, dark-spotted species In: Last PR, editor. Australian catsharks of the genus *Asymbolus* (Carcharhiniformes: Scyliorhinidae) Report 239. Hobart: CSIRO. Division of Marine Research; 1999 p. 19–35.

[37] Naylor G. Chondrichthyan Tree of Life 2015 [10 February 2016]. Available from: http://sharksrays.org/.

[38] Ebert DA, Compagno LJV, Cowley PD. Reproductive biology of catsharks (Chondrichthyes: Scyliorhinidae) off the west coast of southern Africa. ICES Journal of Marine Science: Journal du Conseil. 2006;63(6):1053–65.

[39] Flammang B, Ebert D, Cailliet G. Reproductive biology of deep-sea catsharks (Chondrichthyes: Scyliorhinidae) in the eastern North Pacific. Environ Biol Fishes. 2008;81(1):35–49. 10.1007/s10641-006-9162-9

[40] Trinnie FI, Walker TI, Jones PL, Laurenson LJ. Biennial reproductive cycle in an extensive matrotrophic viviparous batoid: the sandyback stingaree *Urolophus bucculentus* from south-eastern Australia. J Fish Biol. 2012;80(5):1267–91. 10.1111/j.1095-8649.2012.03259.x 22497383

[41] Bridge NF, Mackay D, Newton G. Biology of the ornate angel shark (*Squatina tergocellata*) from the Great Australian Bight. Aust J Mar Freshw Res. 1998;49(7):679–86. 10.1071/MF97075

[42] Clarke MW, Kelly CJ, Connolly PL, Molloy JP. A life history approach to the assessment and management of deepwater fisheries in the northeast Atlantic. J Northwest Atl Fish Sci. 2003;31:401–11.

[43] Last PR. New short-snout members of the skate genus *Dipturus* (Rajoidei:Rajidae) from Australian seas In: Last PR, White WT, Pogonoski JJ, Gledhill DC, editors. Descriptions of new Australian Skates (Batoidea: Rajoidei): CSIRO Marine and Atmospheric Research Paper No. 021; 2008 p. 53–98.

[44] White WT, Last PR. Descriptions of two new species of gummy sharks, genus *Mustelus* (Carcharhiniformes: Triakidae), from Australian waters In: Last PR, White WT, Pogonoski JJ, editors. Descriptions of new Australian chondrichthyans. CSIRO, Hobart: CSIRO Marine and Atmospheric Research Paper No. 022; 2008 p. 189–202.

[45] Cortés E. Life history patterns, demography, and population dynamics In: Carrier JC, Musick JA, Heithaus MR, editors. Biology of sharks and their relatives. Florida: CRC Press; 2004 p. 449–70.

[46] White WT, Platell ME, Potter IC. Relationship between reproductive biology and age composition and growth in *Urolophus lobatus* (Batoidea: Urolophidae). Mar Biol. 2001;138(1):135–47. 10.1007/s002270000436

[47] White WT, Potter IC. Reproductive biology, size and age compositions and growth of the batoid *Urolophus paucimaculatus*, including comparisons with other species of the Urolophidae. Mar Freshw Res. 2005;56(1):101–10. 10.1071/MF04225

[48] Barnett L, Earley R, Ebert D, Cailliet G. Maturity, fecundity, and reproductive cycle of the spotted ratfish, *Hydrolagus colliei*. Mar Biol. 2009;156(3):301–16. 10.1007/s00227-008-1084-y

[49] Moura T, Figueiredo I, Machado PB, Gordo LS. Growth pattern and reproductive strategy of the holocephalan *Chimaera monstrosa* along the Portuguese continental slope. J Mar Biol Assoc U K. 2004;84(04):801–4. 10.1017/S002531540400997Xh

[50] Capapé C, Seck AA, Gueye-Ndiaye A, Diatta Y, Diop M. Reproductive biology of the smoothback angel shark, *Squatina oculata* (Elasmobranchii: Squatinidae), from the coast of Senegal (eastern tropical Atlantic). J Mar Biol Assoc U K. 2002;82(04):635–40. 10.1017/S0025315402005994

[51] Capapé C, Diatta Y, Seck AA, Guelorget O, Souissim J, Zaouali J. Reproduction of the sawback angelshark *Squatina aculeata* (Chondrichthyes:Squatinida) off Senegal and Tunisia. Cybium. 2005;29(2):147–57.

[52] Cailliet GM, Musick JA, Simpfendorfer C, Stevens JD. Ecology and life history characteristics of chondrichthyan fish In: Fowler SL, Cavanagh RD, Camhi M, Burgess GH, Cailliet GM, Fordham SV, et al, editors. Sharks, rays, chimaeras: status of the chondrichthyan fishes: IUCN/SSC Shark Specialist Group; 1990 p. 12–8.

[53] Frisk MG, Miller TJ, Fogarty MJ. Estimation and analysis of biological parameters in elasmobranch fishes: a comparative life history study. Can J Fish Aquat Sci. 2001;58:969–81. 10.1139/cjfas-58-5-969

[54] Treloar MA. Aspects of the life history of skates from southeastern Australia [Ph.D.]. Geelong, Australia: Deakin University; 2008.

[55] Braccini JM, Gillanders BM, Walker TI. Determining reproductive parameters for population assessments of chondrichthyan species with asynchronous ovulation and parturition: piked spurdog (*Squalus megalops*) as a case study. Mar Freshw Res. 2006;57(1):105–19. 10.1071/MF05076

[56] Francis MP, Maolagáin CÓ. Age, growth and maturity of a New Zealand endemic shark (*Mustelus lenticulatus*) estimated from vertebral bands. Mar Freshw Res. 2000;51(1):35–42. 10.1071/MF99012

[57] Kyne PM, Courtney AJ, Bennett MB. Observations on the reproductive biology of three catsharks (Carcharhiniformes: Scyliorhinidae: *Asymbolus* and *Figaro*) from the continental shelf of southern Queensland, Australia. J Mar Biol Assoc U K. 2011;91(Special Issue 06):1157–64. 10.1017/S0025315410001670

[58] Capapé C, Guélorget O, Vergne Y, Reynaud C. Reproductive biology of the blackmouth catshark, *Galeus melastomus* (Chondrichthyes: Scyliorhinidae) off the Languedocian coast (southern France, northern Mediterranean). J Mar Biol Assoc U K. 2008;88(02):415–21. 10.1017/S002531540800060X

[59] Francis M. Distribution and Biology of the New Zealand Endemic Catshark, *Halaelurus dawsoni*. Environ Biol Fishes. 2006;75(3):295–306. 10.1007/s10641-006-0026-0

[60] Castro JI, Bubucis PM, Overstrom NA. The reproductive biology of the chain dogfish, *Scyliorhinus retifer*. Copeia. 1988;1988(3):740–6. 10.2307/1445396

[61] Irvine SB, Daley RK, Graham KJ, Stevens JD. Biological vulnerability of two exploited sharks of the genus *Deania* (Centrophoridae). J Fish Biol. 2012;80(5):1181–206. 10.1111/j.1095-8649.2012.03262.x 22497378

[62] Graham KJ, Daley RK. Distribution, reproduction and population structure of three gulper sharks (*Centrophorus*, Centrophoridae) in south-east Australian waters. Mar Freshw Res. 2011;62(6):583–95. 10.1071/MF10158

[63] Kyne PM, Simpfendorfer CA. A collation and summarization of available data on deepwater chondrichthyans: biodiversity, life history and fisheries.: IUCN Shark Specialist Group; 2007 137 p.

[64] Vooren CM, Chiaramonte GE. Squatina argentina. The IUCN Red List of Threatened Species. Version 2015.2 2006 [26 July 2015]. Available from: www.iucnredlist.org.

[65] Kyne PM, Courtney AJ, Bennett MB. Aspects of reproduction and diet of the Australian endemic skate *Dipturus polyommata* (Ogilby) (Elasmobranchii: Rajidae), by-catch of a commercial prawn trawl fishery. J Fish Biol. 2008;72(1):61–77. 10.1111/j.1095-8649.2007.01655.x

[66] Ruocco NL, Lucifora LO, de Astarloa JMD, Wohler O. Reproductive biology and abundance of the white-dotted skate, *Bathyraja albomaculata*, in the Southwest Atlantic. ICES J Mar Sci. 2006;63(1):105–16. 10.1016/j.icesjms.2005.08.007 ISI:000235095100011.

[67] Richardson AJ, Maharaj G, Compagno LJV, Leslie RW, Ebert DA, Gibbons MJ. Abundance, distribution, morphometrics, reproduction and diet of the Izak catshark. J Fish Biol. 2000;56(3):552–76. 10.1111/j.1095-8649.2000.tb00755.x

[68] IMOS. Ocean Current—Temperature and salinity down to 2000m depth 2015 [cited 2015 10 February 2015.]. Available from: http://oceancurrent.imos.org.au/profiles/map/20110619.html.

[69] Weeks SJ, Bakun A, Steinberg CR, Brinkman R, Hoegh-Guldberg O. The Capricorn Eddy: a prominent driver of the ecology and future of the southern Great Barrier Reef. Coral Reefs. 2010;29(4):975–85. 10.1007/s00338-010-0644-z

[70] Jaine FRA, Rohner CA, Weeks SJ, Couturier LIE, Bennett MB, Townsend KA, et al Movements and habitat use of reef manta rays off eastern Australia: offshore excursions, deep diving and eddy affinity revealed by satellite telemetry. Mar Ecol Prog Ser. 2014;510:73–86. 10.3354/meps10910

[71] Daley RK, Williams A, Green M, Barker B, Brodie P. Can marine reserves conserve vulnerable sharks in the deep sea? A case study of *Centrophorus zeehaani* (Centrophoridae), examined with acoustic telemetry. Deep Sea Research Part II: Topical Studies in Oceanography. 2014;115:127–36. 10.1016/j.dsr2.2014.05.017

[72] Ebert D, Sulikowski J. Preface: Biology of skates. Environ Biol Fishes. 2007;80(2):107–10. 10.1007/s10641-007-9244-3

[73] Ebert DA, Compagno LJV, Cowley PD. Aspects of the reproductive biology of skates (Chondrichthyes: Rajiformes: Rajoidei) from southern Africa. ICES J Mar Sci. 2008;65(1):81–102. 10.1093/icesjms/fsm169 CCC:000252523100010.

[74] Frisk MG, Miller TJ, Fogarty MJ. The population dynamics of little skate *Leucoraja erinacea*, winter skate *Leucoraja ocellata*, and barndoor skate *Dipturus laevis*: predicting exploitation limits using matrix analyses. ICES J Mar Sci. 2002;59:576–82. 10.1006/jmsc.2002.1177

[75] Braccini JM, Gillanders BM, Walker TI, Tovar-Avila J. Comparison of deterministic growth models fitted to length-at-age data of the piked spurdog (*Squalus megalops*) in south-eastern Australia. Mar Freshw Res. 2007;58(1):24–33. 10.1071/MF06064

[76] Braccini JM, Gillanders BM, Walker TI. Sources of variation in the feeding ecology of the piked spurdog (*Squalus megalops*): implications for inferring predator–prey interactions from overall dietary composition. ICES Journal of Marine Science: Journal du Conseil. 2005;62(6):1076–94. 10.1016/j.icesjms.2005.04.004

[77] Simpfendorfer CA, Milward NE. Utilisation of a tropical bay as a nursery area by sharks of the families Carcharhinidae and Sphyrnidae. Environ Biol Fishes. 1992;37(4):337–45. 10.1007/bf00005200

[78] Wetherbee BM, Cortes E, Bizzarro JJ. Food consumption and feeding habits In: Carrier JC, Musick J, Heithaus MR, editors. Biology of sharks and their relatives Second edtion Boca Raton, Fl.: CRC Press; 2012 p. 239–64.

[79] Graham KJ, Andrew NL, Hodgson KE. Changes in relative abundance of sharks and rays on Australian South East Fishery trawl grounds after twenty years of fishing. Mar Freshw Res. 2001;52(4):549–61. 10.1071/MF99174

[80] Pogonoski J, Pollard D. *Squatina albipunctata* The IUCN Red List of Threatened Species. Version 2015.2 2003 [14 July 2015]. Available from: www.iucnredlist.org.

[81] Last PR, Marshall LJ. *Urolophus bucculentus* The IUCN Red List of Threatened Species. Version 2015.2. 2006 [14 July 2015]. Available from: www.iucnredlist.org.

[82] Kynoch RJ, Fryer RJ, Neat FC. A simple technical measure to reduce bycatch and discard of skates and sharks in mixed-species bottom-trawl fisheries. ICES Journal of Marine Science: Journal du Conseil. 2015 10.1093/icesjms/fsv037

