# Supplementary material for: Deepwater Chondrichthyan Bycatch of the Eastern King Prawn Fishery in the Southern Great Barrier Reef, Australia
Source: PLoS One. 2016 May 24;11(5):e0156036. doi: 10.1371/journal.pone.0156036 (PMC4878763; doi:10.1371/journal.pone.0156036)
Supplement: S1 Table — Source: A. Great Barrier Reef Marine Park (Chin et al. 2010); B. Eastern King Prawn fishery in the Great Barrier Reef, Department of Agriculture and Fishery Observer Program 2005–2010(Pears et al. 2012); C. Eastern King Prawn fishery in the Swain Reefs area of the Great Barrier Reef (the present study). Australian endemic: if endemic it is stated if restricted to waters offshore from Queensland-New South Wales (QLD-NSW) or Queensland (QLD). IUCN Status from the IUCN Red List of Threatened Species http://www.iucnredlist.org. (DOCX) [file pone.0156036.s001.docx]

**S1 Table. Deepwater chondrichthyans known to occur in the Great Barrier Reef Marine Park**. Source: A. Great Barrier Reef Marine Park ([Chin et al., 2010](#_ENREF_1)); B. Eastern King Prawn fishery in the Great Barrier Reef, Department of Agriculture and Fishery Observer Program 2005-2010 ([Pears et al., 2012](#_ENREF_4)); C. Eastern King Prawn fishery in the Swain Reefs area of the Great Barrier Reef (the present study). Australian endemic: if endemic, it is stated whether it is restricted to waters offshore from Queensland-New South Wales (QLD-NSW) or Queensland (QLD). IUCN Status from the IUCN Red List of Threatened Species: Data Deficient DD, Least Concern LC, Near Threatened NT, Vulnerable VU, Endangered EN Source: <http://www.iucnredlist.org>.

| Family | Species | Common name | GBRMP^A^ | EKP^B^ | EKP^C^ | Australian endemic | IUCN Status |
| --- | --- | --- | --- | --- | --- | --- | --- |
| Chimaeridae | *Chimaera macrospina* | Longspine chimaera | X |  |  | Yes | LC |
|  | *Chimaera obscura* | Shortspine chimaera | X |  |  | QLD-NSW | DD |
|  | *Hydrolagus lemures* | Blackfin ghostshark | X | X | X | Yes | LC |
|  | *Hydrolagus marmoratus* | Marbled ghostshark | X |  |  | QLD-NSW | LC |
| Hexanchidae | *Heptranchias perlo* | Sharpnose sevengill shark | X |  |  | No | NT |
|  | *Hexanchus griseus* | Bluntnose sixgill shark | X |  |  | No | NT |
|  | *Hexanchus nakamurai* | Bigeye sixgill shark | X |  |  | No | DD |
| Echinorhinidae | *Echinorhinus cookei^a^* | Prickly shark | X |  |  | No | NT |
| Squalidae | *Squalus albifrons* | Eastern highfin spurdog | X |  |  | QLD-NSW | DD |
|  | *Squalus grahami* | Eastern longnose spurdog | X |  |  | QLD-NSW | NT |
|  | *Squalus megalops* | Piked spurdog |  |  | X | No | NT |
|  | *Squalus montalbani* | Philippine spurdog | X |  |  | No | VU |
|  | *Squalus notocaudatus* | Bartail spurdog | X |  |  | QLD | DD |
| Centrophoridae | *Centrophorus moluccensis* | Endeavour dogfish | X |  |  | No | NT |
|  | *Centrophorus niaukang* | Taiwan gulper shark | X |  |  | No | Not assessed |
| Etmopteridae | *Etmopterus brachyurus^a^* | Short-tail lanternshark | X |  |  | No | DD |
|  | *Etmopterus dianthus* | Pink lanternshark | X |  |  | No | LC |
|  | *Etmopterus dislineatus* | Lined lanternshark | X |  |  | QLD | LC |
|  | *Etmopterus lucifer* | Blackbelly lanternshark | X |  |  | No | LC |
| Dalatiidae | *Dalatias licha* | Black shark | X |  |  | No | NT |
| Squatindae | *Squatina albipunctata* | Eastern angelshark | X |  | X | QLD-NSW | VU |
| Pristophoridae | *Pristiophorus delicatus* | Tropical sawshark | X |  | X | QLD | LC |
| Narcinidae | *Narcine nelsoni* | Eastern numbfish | X |  |  | QLD | LC |
| Torpedinidae | *Torpedo macneilli* | Short-tail torpedo ray | X |  |  | Yes | DD |
|  | *Torpedo tokionis* | Longtail torpedo ray | X |  |  | No | DD |
| Arhynchobatidae | *Insentiraja laxipella* | Eastern looseskin skate | X |  |  | QLD | DD |
|  | *Notoraja ochroderma* | Pale skate | X |  |  | QLD | DD |
|  | *Pavoraja mosaica* | Mosaic skate | X |  |  | QLD | LC |
|  | *Pavoraja pseudonitida* | False peacock skate | X |  |  | QLD | LC |
| Rajidae | *Dipturus apricus* | Pale tropical skate | X | X | X | QLD | LC |
|  | *Dipturus melanospilus* | Blacktip skate | X |  |  | QLD-NSW | DD |
|  | *Dipturus polyommata* | Argus skate | X | X | X | QLD | LC |
|  | *Dipturus queenslandicus* | Queensland deepwater skate | X |  |  | QLD | DD |
|  | *Dipturus wengi* | Weng's skate | X |  |  | Yes | LC |
| Anacanthobatidae | *Sinobatis filicauda* | Eastern leg skate | X |  |  | QLD | DD |
| Plesiobatidae | *Plesiobatis daviesi* | Giant stingaree | X |  |  | No | LC |
| Urolophidae | *Urolophus bucculentus^b^* | Sandyback stingaree | X | X | X | Yes | VU |
|  | *Urolophus piperatus* | Coral Sea stingaree | X |  | X | QLD | LC |
| Hexatrygonidae | *Hexatrygon bickelli* | Sixgill stingray | X |  |  | No | LC |
| Myliobatidae | *Myliobatis hamlyni* | Purple eagle ray | X |  |  | Yes | EN |
| Brachaeluridae | *Brachaelurus colcloughi^c^* | Colcough’s shark | X | X | X | QLD-NSW | VU |
| Orectolobidae | *Orectolobus maculatus^c^* | Spotted wobbegong | X | X | X | No | LC |
| Scyliorhinidae | *Apristurus australis* | Pinocchio catshark | X |  |  | Yes | DD |
|  | *Apristurus longicephalus* | Smoothbelly catshark | X |  |  | No | LC |
|  | *Apristurus platyrhynchus* | Bigfin catshark | X |  |  | No | LC |
|  | *Asymbolus pallidus* | Pale spotted catshark | X | X | X | QLD | LC |
|  | *Cephaloscyllium signourum* | Flagtail swellshark | X |  |  | No | DD |
|  | *Cephaloscyllium variegatum* | Saddled swellshark | X | X | X | QLD-NSW | NT |
|  | *Cephaloscyllium zebrum* | Narrowbar swellshark | X |  |  | QLD | DD |
|  | *Figaro striatus* | Northern sawtail shark | X |  |  | QLD | DD |
|  | *Galeus gracilis* | Slender sawtail shark | X |  |  | Yes | DD |
|  | *Parmaturus bigus* | Short-tail catshark | X |  |  | QLD | DD |
| Pseudotriakidae | *Pseudotriakis microdon* | False catshark | X |  |  | No | LC |
| Triakidae | *Hemitriakis abdita* | Darksnout houndshark | X |  |  | QLD | DD |
|  | *Iago garricki* | Longnose houndshark | X |  |  | No | LC |
|  | *Mustelus walkeri* | Eastern spotted gummy shark | X | X | X | QLD | DD |
| Carcharhinidae | *Carcharhinus altimus* | Bignose shark | X |  |  | No | DD |

^a^additional deepwater species in GBRMP ([Last and Stevens, 2009](#_ENREF_3))

^b^*Urolophus flavomosaicus* previously recorded from GBRMP are likely to be *U. bucculentus*

^c^we consider these shelf species, based on Last and Stevens ([2009](#_ENREF_3)).

**References**

Chin A, Kyne PM, Walker TI, McAuley RB. An integrated risk assessment for climate change: analysing the vulnerability of sharks and rays on Australia's Great Barrier Reef. Glob Change Biol. 2010;16(7):1936-53. doi: 10.1111/j.1365-2486.2009.02128.x.

Last PR, Stevens JD. Sharks and rays of Australia. 2nd ed. Melbourne: CSIRO Publishing; 2009. 644 p.

Pears RJ, Morison AK, Jebreen EJ, Dunning MC, Pitcher CR, Courtney AJ, et al. Ecological risk assessment of the East Coast Otter Trawl Fishery in the Great Barrier Reef Marine Park: Summary report. Townsville: 2012.
