# Supplementary material for: Deepwater Chondrichthyan Bycatch of the Eastern King Prawn Fishery in the Southern Great Barrier Reef, Australia
Source: PLoS One. 2016 May 24;11(5):e0156036. doi: 10.1371/journal.pone.0156036 (PMC4878763; doi:10.1371/journal.pone.0156036)
Supplement: S2 Table — All lengths are for LST unless otherwise specified as disc width (WD). The percent of shelf bycatch is by abundance. (DOCX) [file pone.0156036.s002.docx]

**S2 Table. Observed shelf shark and ray species by sex, abundance and length.** All lengths are for *L*_ST_ unless otherwise specified as disc width (*W*_D_)_._ The percent of shelf bycatch is by abundance.

| Scientific name | Common name | Male  (n) | Female  (n) | Total  (n) | Depth range  (m) | Length range  (*L*_ST_ mm) | % Shelf species bycatch  (n) |
| --- | --- | --- | --- | --- | --- | --- | --- |
| *Hemigaleus australiensis* | Australian weasel shark | 15 | 31 | 46 | 117–137 | 450–820 | 32.4 |
| *Brachaelurus colcloughi* | Colclough’s shark | 17 | 10 | 27 | 128–157 | 410–880 | 19.0 |
| *Aptychotrema rostrata* | Eastern shovelnose ray | 9 | 9 | 18 | 123–150 | 490–900 | 12.7 |
| *Carcharhinus coatesi* | Whitecheek shark | 8 | 7 | 15 | 128–137 | 520–600 | 10.6 |
| *Carcharhinus plumbeus* | Sandbar shark | 7 | 6 | 13 | 135–196 | 720–1620 | 9.2 |
| *Orectolobus maculatus* | Spotted wobbegong | 5 | 6 | 11 | 128–242 | 820–1420 | 7.7 |
| *Gymnura australis* | Australian butterfly ray | 2 | 3 | 5 | 117–137 | 520–840 *W*_D_ | 3.5 |
| *Carcharhinus amblyrhynchoides* | Graceful shark | 1 | 1 | 2 | 146–152 | 1490–1760 | 1.4 |
| *Carcharhinus falciformis* | Silky shark | 0 | 1 | 1 | 139 | 1230 | 0.7 |
| *Carcharhinus sorrah* | Spot-tail shark | 1 | 0 | 1 | 133 | 1310 | 0.7 |
| *Chiloscyllium punctatum* | Grey carpetshark | 0 | 1 | 1 | 126 | 520 | 0.7 |
| *Dasyatis thetidis* | Black stingray | 0 | 1 | 1 | 190 | 400 *W*_D_ | 0.7 |
| *Rhynchobatus palpebratus* | Eyebrow wedgefish | 0 | 1 | 1 | 136 | 1200 | 0.7 |
| Total abundance shelf species | |  |  | 142 |  |  |  |

Note: Any shelf sharks and rays landed were identified, sexed, measured for stretched total length (*L*_ST_) or disc width (*W*_D_), photographed and returned to the sea. Species identifications were confirmed by one of the authors (WW) from the photographs. Six additional shelf species were recorded in this study that had not previously been reported from the deepwater EKP fishery within the GBRMP: *Hemigaleus australiensis*, *Carcharhinus amblyrhynchoides*, *Carcharhinus falciformis*, *Carcharhinus sorrah*, *Dasyatis thetidis* and *Rhynchobatus palpebratus* ([Pears et al., 2012](#_ENREF_4)). The maximum length and depth of *Brachaelurus colcloughi* was extended from 770 mm to 880 mm and 100 m to 157 m, respectively ([Kyne, 2008](#_ENREF_2); [Last and Stevens, 2009](#_ENREF_3)). The known maximum depth of three other shelf species was also extended: *Orectobolus maculatus* from 218 m to 242 m, *Chiloscyllium punctatum* from at least 85 m to 126 m, and *Rhynchobatus palpebratus* from 60 m to 136 m ([Last and Stevens, 2009](#_ENREF_3)).

**References**

Kyne PM. Chondrichthyans and the Queensland East Coast Trawl Fishery: Bycatch reduction, biology, conservation status and sustainability [Doctor of Philosophy]. Brisbane.: University of Queensland; 2008.

Last PR, Stevens JD. Sharks and rays of Australia. 2nd ed. Melbourne: CSIRO Publishing; 2009. 644 p.

Pears RJ, Morison AK, Jebreen EJ, Dunning MC, Pitcher CR, Courtney AJ, et al. Ecological risk assessment of the East Coast Otter Trawl Fishery in the Great Barrier Reef Marine Park: Summary report. Townsville: 2012.
