# Supplementary material for: Deepwater Chondrichthyan Bycatch of the Eastern King Prawn Fishery in the Southern Great Barrier Reef, Australia
Source: PLoS One. 2016 May 24;11(5):e0156036. doi: 10.1371/journal.pone.0156036 (PMC4878763; doi:10.1371/journal.pone.0156036)
Supplement: S3 Table — The GN numbers refer to samples with NADH2 sequences available as part of the Chondrichthyan Tree of Life project (http://sharksrays.org/). (DOCX) [file pone.0156036.s003.docx]

**S3 Table. Genetic samples for deepwater chondrichthyan specimens collected from Swain Reefs Eastern King Prawn Fishery.** The GN numbers refer to samples with NADH2 sequences available as part of the Chondrichthyan Tree of Life project (<http://sharksrays.org/>).

| Species name | Genetic sample number |
| --- | --- |
| *Squalus megalops* | GN15689; GN15690 |
| *Asymbolus pallidus* | GN11021; GN11022; GN11023; GN11024; GN11025 |
| *Urolophus piperatus* | GN11026; GN11027; GN11028; GN15673; GN15674 |
| *Squatina albipunctata* | GN15676; GN15677 |
| *Dipturus apricus* | GN15678; GN15679 |
| *Cephaloscyllium variegatum* | GN15669 |
| *Dipturus polyommata* | GN11017; GN11018; GN11019; GN11020; GN15691; GN15692; GN15693; GN15694 |
| *Mustelus walkeri* | GN15680; GN15681; GN15682; GN15683; GN15684 |
| *Hydrolagus lemures* | GN7388 |
| *Urolophus bucculentus* | GN15685; GN15686; GN15687 |
