# Supplementary material for: Deepwater Chondrichthyan Bycatch of the Eastern King Prawn Fishery in the Southern Great Barrier Reef, Australia
Source: PLoS One. 2016 May 24;11(5):e0156036. doi: 10.1371/journal.pone.0156036 (PMC4878763; doi:10.1371/journal.pone.0156036)
Supplement: S4 Table — Australian endemic: if endemic it is stated if restricted to waters offshore from Queensland-New South Wales (QLD-NSW) or Queensland (QLD). Source: Last and Stevens, 2009. (DOCX) [file pone.0156036.s004.docx]

**S4 Table. Depth and distribution range of deepwater chondrichthyans observed from Swain Reefs Eastern King Prawn Fishery.** Australian endemic: if endemic, it is stated whether it is restricted to waters offshore from Queensland–New South Wales (QLD–NSW) or Queensland (QLD). Source: Last and Stevens, 2009.

| Species | Depth range (m) | Distribution range | Australian endemic |
| --- | --- | --- | --- |
| *Squalus megalops* | 0–732 | Atlantic to Indo-West Pacific: in Australia south of Whitsundays continuous to the north west shelf of Western Australia | No |
| *Dipturus polyommata* | 135–320 | Rockhampton to Townsville, probably further north | Yes- QLD |
| *Asymbolus pallidus* | 225–400 | Swain Reefs to Cairns | Yes- QLD |
| *Mustelus walkeri* | 50–400 | Moreton Island to Hinchinbrook, possibly further north and south | Yes- QLD |
| *Urolophus piperatus* | 170–370 | Moreton Island to Cairns | Yes- QLD |
| *Pristiphorus delicatus* | 245–405 | Rockhampton to Cairns | Yes- QLD |
| *Hydrolagus lemures* | 200–700 | Cairns continuous to Scott Reef , Western Australia | Yes |
| *Urolophus bucculentus* | 65–265 | Beachport South Australia to Stradbroke Island, Queensland- extended to Swain Reefs, Queensland | Yes |
| *Squatina albipunctata* | 35–415 | Lakes Entrance, Victoria to Cairns, Queensland | Yes |
| *Dipturus apricus* | 240–695 | Broken Bay, New South Wales to Marion Plateau, Queensland | Yes |
| *Cephaloscyllium variegatum* | 115–605 | Tathra, New South Wales to Rockingham Bay, Queensland | Yes |

**References**

Last PR, Stevens JD. Sharks and rays of Australia. 2nd ed. Melbourne: CSIRO Publishing; 2009. 644 p.
