# Supplementary material for: Deepwater Chondrichthyan Bycatch of the Eastern King Prawn Fishery in the Southern Great Barrier Reef, Australia
Source: PLoS One. 2016 May 24;11(5):e0156036. doi: 10.1371/journal.pone.0156036 (PMC4878763; doi:10.1371/journal.pone.0156036)
Supplement: S5 Table — The growth completion rate k is the von Bertalanffy growth function. Note: M. walkeri male age at maturity was based on the largest adolescent male and the one mature male collected. Source: Rigby et al. 2105, Rigby et al. 2016. (DOCX) [file pone.0156036.s005.docx]

**S5 Table. Age and growth data for *Dipturus polyommata*, *Squalus megalops* and *Mustelus walkeri*.** The growth completion rate *k* is the von Bertalanffy growth function. Note: *M. walkeri* male age at maturity was based on the largest adolescent male and the one mature male collected. Source: Rigby *et al.* 2105, Rigby *et al.* 2016.

|  | Age at maturity  (years) | | Observed Longevity | | VBGF *k* (year^-1^) |
| --- | --- | --- | --- | --- | --- |
|  | **Male** | **Female** | **Male** | **Female** | **Sexes combined** |
| *Squalus megalops* | 12.6 | 19.1 | 18 | 25 | 0.003 |
| *Dipturus polyommata* | 4.0 | 5.1 | 10 | 10 | 0.208 |
| *Mustelus walkeri* | 7-9 | 10-14 | 9 | 16 | 0.033 |

**References**

Rigby CL, Daley RK, Simpfendorfer CA. Comparison of life histories of two deepwater sharks from eastern Australia: the piked spurdog and the Philippine spurdog. Marine and Freshwater Research. 2015. doi: 10.071/MF15176.

Rigby CL, White WT, Smart JJ, Simpfendorfer CA. Life histories of two deep-water Australian endemic elasmobranchs: Argus skate *Dipturus polyommata* and eastern spotted gummy shark *Mustelus walkeri*. J Fish Biol. 2016;88:1149-74. doi: 10.1111/jfb.12891.
